# A practical overview of CT and MRI features of developmental, inflammatory, and neoplastic lesions of the sphenoid body and clivus

**DOI:** 10.1007/s00234-022-02986-x

**Published:** 2022-06-03

**Authors:** Cosimo Nardi, Davide Maraghelli, Michele Pietragalla, Elisa Scola, Luca Giovanni Locatello, Giandomenico Maggiore, Oreste Gallo, Maurizio Bartolucci

**Affiliations:** 1 Department of Experimental and Clinical Biomedical Sciences, Radiodiagnostic Unit N. 2, University of Florence - Azienda Ospedaliero-Universitaria Careggi, Largo Brambilla 3, 50134 Florence, Italy; 2 Department of Neuroradiology, Careggi University Hospital, Largo Piero Palagi 1, 50134 Florence, Italy; 3 Department of Otorhinolaryngology, Careggi University Hospital, Via Taddeo Alderotti, 50139 Florence, Italy; 4 Department of Experimental and Clinical Medicine, University of Florence - Azienda Ospedaliero-Universitaria Careggi, Largo Brambilla 3, 50134 Florence, Italy; 5 Department of Radiology, Azienda USL Toscana Centro, Santo Stefano Hospital, Via Suor Niccolina Infermiera, 20/22, 59100 Prato, Italy

**Keywords:** Sphenoid bone, Clivus, Cancer, Computed tomography, Magnetic resonance imaging

## Abstract

The sphenoid bone is an unpaired bone that contributes to the formation of the skull base. Despite the enormous progress in transnasal endoscopic visualisation, imaging techniques remain the cornerstones to characterise any pathological condition arising in this area. In the present review, we offer a bird’s-eye view of the developmental, inflammatory, and neoplastic alterations affecting the sphenoid body and clivus, with the aim to propose a practical diagnostic aid for radiologists based on clinico-epidemiological, computed tomography, and magnetic resonance imaging features.

## Introduction

The sphenoid (from the Greek “sphenoeides”, “wedgelike”) bone is a butterfly-shaped, median single structure that articulates with frontal, ethmoid, zygomatic, parietal, temporal, occipital, palatine, and vomer bones: it represents the key intersection of the anterior, middle, and posterior cranial fossa [1]. It is formed by the greater and lesser wings, the medial and lateral pterygoid processes, and by a variably pneumatized central body: the latter is located between the greater wings, and it houses the two sphenoidal sinuses along with a depression named “sella turcica” (from the Latin, “Turkish saddle”). The sphenoid bone is surrounded by many critical neurovascular structures such as the pituitary gland; the C3-C4-C5 segments of the internal carotid artery; the optic nerves and chiasm; cranial nerves III, IV, V1, V2, and VI; the sphenopalatine ganglion and artery; the cavernous sinus; and the ventral brainstem. It also contributes to the formation of fissures and foramina of the skull base such as the optic canal, superior and inferior orbital fissures, and foramen rotundum, lacerum, spinosum, and ovale; in addition, it contains important radiological and surgical landmarks such as the optic and maxillary struts and the Vidian canal (from the Florentine anatomist, Vidus Vidius) [2]. The clivus is the inclined midline surface of the skull base, just anterior to the foramen magnum, and which is formed by the sphenoid body and the basilar part of the occipital bone (basiocciput) [1].

The aim of this review is to summarise the clinico-epidemiological, computed tomography (CT), and magnetic resonance imaging (MRI) features of a wide variety of lesions involving the sphenoid body and the clivus. Such features may guide radiologists and skull base surgeons towards the most appropriate diagnostic and/or therapeutic approaches to the sphenoid/clival lesions.

## Imaging techniques, classification, and clinico-epidemiological features of sphenoid/clival lesions

Radiological investigation techniques for the assessment of any lesion involving this area are nowadays represented by CT and MRI, which play a complementary role in the study of the whole skull base. The former is extremely useful in the identification of the sphenoid bone profile, erosions, sclerosis, lysis, intrasinusal content, and extra-sphenoid extension of disease. MRI is instead crucial to define both intracranial and extracranial soft tissue invasions, to evaluate the possible involvement of cranial nerves, and to further characterise intrasinusal contents [3]. Skull X-ray projections are currently considered an outdated examination with a significantly lower accuracy than CT and MRI; therefore, radiographs have no more indications in skull base radiology [4]. While FDG-PET/CT can be used as a support to CT and MRI, especially for staging purposes, its role in sphenoidal conditions falls beyond the scope of this paper.

From an epidemiological point of view, sphenoid lesions are quite uncommon, and they are classically classified into developmental, inflammatory, and neoplastic pathologies, each of them with extremely variable imaging patterns [5]. Traumatic sphenoid and/or clival lesions, which we have excluded from this classification, represent an emergency issue that is invariably associated with other craniofacial injuries and will not be further discussed. Benign and malignant tumoural pathologies primarily arising from the sphenoid body and clivus are rare with an estimated incidence rate of < 50 cases per 100,000 inhabitants [6]. This localization is also uncommon for inflammatory diseases, if we exclude secondary involvement of the sphenoid sinus in both acute and chronic rhinosinusitis: only 20% of all paranasal mycetomas, 1–2% of all paranasal mucoceles, and 2% of all cases of skull base osteomyelitis involve the sphenoid bone [7–12]. Regarding developmental alterations, it is not easy to define their prevalence since they often remain asymptomatic lifelong. Arrested pneumatisation of the sphenoid sinus is believed to be the most frequent developmental alteration; ecchordosis physaliphora is still one of the more frequent alterations since it can be found in 0.4–2% of autopsies [13]. Clinical manifestations of sphenoid/clival lesions are variable. Developmental lesions and haemangiomas are often incidental findings, whereas inflammatory lesions or tumours are almost always symptomatic. They may present with headache (40% of symptomatic cases), visual disturbances, and cranial neuropathies due to the critical neurovascular relationships with the aforementioned structures [14, 15]. Headache is typically described as deeply located and retro-orbital; visual disturbances can be grouped into three neurologically distinct syndromes: the sphenocavernous syndrome (deficits of cranial nerves III, IV, VI, V1, and V2, with or without the involvement of the optic nerve and the oculosympathetic fibres), an isolated abducens palsy (for only cranial nerve VI involvement, which runs in the narrow Dorello’s canal), and an isolated loss of visual function [14]. Ominous signs and symptoms such as facial numbness, facial pain, epistaxis, or anosmia are instead much rarer [14]. In Tables 1, 2 and 3 we have summarised the main clinical manifestations of each pathology.

**Table 1:** Clinico-epidemiological features of developmental and inflammatory sphenoid bone lesions. *M* male. *F* female. *CN* cranial nerve

Developmental and inflammatory sphenoid lesions	Prevalence	Age (decade) of peak incidence of onset	Gender predilection	Clinical manifestations
Ecchordosis physaliphora [13]	Found in about 0.4–2% of autopsies	Congenital	M = F	Usually asymptomatic and does not require any treatment
Neurenteric cyst [21, 22]	Intracranial localization is rare (0.15–0.35% of all intracranial tumours); clivus localization is described in very few sporadic cases	III–IV decade	M = F	Often symptomatic with headache and diplopia
Arrested pneumatization [11, 24]	2% in the general population; 10% in patients with blood-red cell diseases (sickle cell anaemia-thalassemia)	Congenital and developmental lesion	M = F	Headache and obstructive symptoms due to alterations of normal sinus drainage
Epidermoid cyst [31, 32]	Located in the head-neck area in 7% of cases	Often congenital. It can also occur in adult age for metaplasia or trauma	M = F	Symptoms are very rare. They generally occur in III-V decade and include visual disturbances (compression of CN II), infection due to adjacent sinusitis, and pituitary apoplexy in case of sellar extension
Fibrous dysplasia [37]	The skull is involved in 10–30% and 50% of monostotic and polyostotic forms respectively. Sphenoid is one of the main craniofacial areas	Congenital	M = F	Supraorbital headache is the most frequent symptom
Fungus ball (mycetoma) [7]	Typically affects the maxillary sinus. The sphenoid sinus is involved in about 20% of paranasal fungus balls	V-VI decade	M:F ratio = 3:7	Frontal, retro-orbital, and occipital headache. Visual disturbances due to the CN II and VI impairment. Rarely asymptomatic
Mucocele [8, 9]	Sphenoid sinus is a rare localization for mucocele, accounting for 1–2% of all paranasal mucoceles	Any age	M = F	Posterior headache is the most common symptom. Visual disturbances may be associated when mucocele compresses CN II into the orbit and/or CN III, IV, and VI into the cavernous sinus
Osteomyelitis [10, 12]	< 2% and around 10% of all osteomyelitis in developed and developing countries, respectively	Any age	M:F ratio = 2:1	Headache is commonly the only initial symptom

**Table 2:** Clinico-epidemiological features of sphenoid bone benign tumours. *M* male. *F* female

Sphenoid benign tumour	Prevalence	Age (decade) of peak incidence of onset	Gender predilection	Clinical manifestations
Haemangioma [103]	< 1% of all bone tumours. < 10 cases are described for clival localizations	IV decade	No M:F ratio reported. Most frequent in women	Often asymptomatic. Headache, sight loss, and compression of the carotid artery or cavernous sinus if haemangioma is large
Ossifying fibroma [60]	No prevalence reported	I-III decade	M = F	Diplopia and headache
Pituitary adenoma [104, 105]	10% of intracranial tumours	Prolactinoma: III decade. Non-functioning adenoma: > V decade	Prolactinoma: M:F ratio = 1:5–14 Non-functioning adenoma M:F ratio = 3:1	Invasive forms cause headache and visual disturbances due to the involvement of the optic chiasm and cavernous sinus. Hormonal disorders in case of functioning adenomas
Sinonasal papilloma [71]	Sphenoid localization represents 5–10% of all inverted papillomas	V–VI decade	M:F ratio = 3:1	Non-specific disorders. The most common symptom is headache

**Table 3:** Clinico-epidemiological features of malignant tumours that primarily or secondarily involve the sphenoid bone. *M* male. *F* female. *CN* cranial nerve

Sphenoid malignant tumour	Prevalence	Age (decade) of peak incidence of onset	Gender predilection	Clinical manifestations
Chordoma [77, 78]	6% of all primary bone tumours. Incidence: 0.08 per 100,000	III–V decade	M = F	CN VI palsy-related. Diplopia and headache are the most common initial disorders
Nasopharyngeal carcinoma [82]	Sphenoid sinus is invaded in 20% of cases	V–VII decade	M:F ratio = 5:2	Symptoms of the primary mass such as nasal obstruction, epistaxis, and conductive hearing loss due to the Eustachian tube obstruction
Neuroendocrine tumour [89, 90]	5% of all sinonasal malignancies. The “poorly differentiated” form is the most frequent sphenoid neuroendocrine tumour	V–VI decade	M = F	Headache, epistaxis, and visual disturbances. A paraneoplastic syndrome due to the ectopic hormone production is rare
Lymphoma [15, 91]	Primary sphenoid sinus lymphoma is very rare (only 20 cases described)	> V decade	M:F ratio = 3:1	Non-specific symptoms including recurrent sinusitis, nasal discharge, headache, and sight loss
Multiple Myeloma/plasmacytoma [98, 106]	Head-neck localization is very rare. 75–80% of extramedullary plasmacytomas arise from the aerodigestive tract. Plasmacytomas of the sphenoid sinus account for 1.6% of all solitary extramedullary plasmacytomas	VII–VIII decade	M:F ratio = 1.5:1	Headache and CN II, III, IV, and VI palsies are the most frequent manifestations
Sphenoid bone metastases [101]	< 1% of all intracranial tumours	VI decade	M:F ratio = 3:1	Diplopia and headache represent the most prevalent symptoms

## The role of radiologists in the evaluation of sphenoid lesions

Radiologists play a central role in the diagnostic assessment of every sphenoid lesion since only a limited view of the anterior walls of its sinusal cavities can be performed in the office setting, even with modern transnasal endoscopy. Furthermore, its unfavourable anatomical location sometimes requires extensive surgical dissection in order to obtain a tissue sample by transnasal biopsy [4, 16, 17].

Multidisciplinary cooperation among radiologists, pathologists, neurosurgeons, and otolaryngologists is thus essential. Diagnostic imaging plays a central role in the process of differential diagnosis, especially between inflammatory and neoplastic lesions. The radiologist must provide diagnostic hypotheses for each condition by the identification of clues and features of malignancies [18]. Furthermore, some benign sphenoid bone alterations — such as neurenteric cyst, haemangioma, arrested pneumatization, or fibrous dysplasia — may be diagnosed only on the basis of their imaging features. For this reason, some authors have cleverly spoken of “do-not-touch” sphenoid lesions for these four entities [16]. Table 4 illustrates the most relevant CT and MRI features of sphenoid body and clival diseases.

**Table 4:** CT and MRI features of the sphenoid bone lesions. Differential diagnoses are discussed in the text. *CT* computed tomography. *MRI* magnetic resonance imaging. *DWI* diffusion weighted imaging. *ADC* apparent diffusion coefficient. *CE* contrast enhancement. *SI* signal intensity

Sphenoid lesion	CT	MRI-T1W	MRI-T2W	MRI-CE-T1	DWI	Differential diagnoses
Ecchordosis physaliphora	Well-defined bony clival defect with cortical preservation and a bony “stalk” at its base	Low SI	High SI	No	Facilitated diffusion	Chordoma, dermoid cyst, arachnoid cyst
Neurenteric cyst	Lytic lesion with cortical preservation and variable density based on protein content	Low SI. High SI if high protein content	High SI	No	Facilitated diffusion	Dermoid cyst, “white epidermoid”, arachnoid cyst, nerve sheath tumours
Arrested pneumatization	Non-expansive area with osteosclerotic margins and linear calcifications	High SI for intralesional foci of fat Low SI for intralesional calcifications	Medium–high SI	No	Facilitated diffusion	Fibrous dysplasia, ossifying fibroma, chordoma, chondrosarcoma, osteomyelitis, metastases, lipoma, haemangioma
Epidermoid cyst	Hypodense. Hyperdense in case of protein deposits. A scalloped or lobulated sclerotic rim is pathognomonic	Low SI High SI in case of protein deposits	High SI	No. A thin peripheral rim of CE may sometimes be found	Restricted diffusion	Arachnoid cyst, abscess, dermoid cysts, neurenteric cyst, mucocele
Fibrous dysplasia	Different specific patterns: ground-glass, homogeneously dense, and cystic patterns	Low-to-intermediate SI	Variable SI	No. Heterogenous CE in case of active disease	Facilitated diffusion	Skull base malignancies, Paget disease, intraosseous meningioma, ossifying fibroma
Fungus ball	Hyperdense mass with calcifications	High SI	Very low or “dark” SI	Sinus mucosa enhanced. No CE for the intraluminal content	Hypointensity on both DWI b_1000_ and ADC map	Mucocele, sinonasal mucosal melanoma
Mucocele	Sinus opacification with variable-density content. Bony wall expansion and focal resorption	Low SI High SI if rich in proteins	High SI Low SI if rich in proteins	The periphery may enhance, but not the central core	Variable. ADC values are very low (< 0.5 × 10^−3^ mm^2^/s) if mucocele is rich in viscid secretions	Fungus ball, sphenoid mucus retention cyst, sphenoid sinus inverted papilloma
Osteomyelitis	No specific pattern. Bony sclerosis and/or erosion	Low SI	High SI	Enhancing soft tissue mass	High SI on DWI. Low ADC values but higher than malignant lesions	Sphenoid/skull base primary and secondary malignancies, especially nasopharyngeal carcinoma
Haemangioma	Expansive, well-circumscribed area of bony rarefaction with the typical “sunburst appearance”	“Mottled” and heterogeneously high SI	“Mottled” and heterogeneously high SI	Marked CE	Facilitated diffusion	Arrested pneumatization, fibrous dysplasia, lipoma, multiple myeloma
Ossifying Fibroma	Expansive mass with a thin sclerotic shell and possible calcifications	Low SI	Low SI. Mixed low–high SI in case of intralesional cysts	Moderate-high CE, usually heterogeneous	Variable DWI and ADC. Generally, no restricted diffusion	Fibrous dysplasia, arrested pneumatization, osteoma, osteosarcoma, chondrosarcoma; if calcified: lymphoma and sinonasal melanoma
Pituitary adenoma	Hypodense mass with cystic, osteolytic, and/or haemorrhagic areas	Medium–low SI	Medium-slightly high SI	Homogeneous CE	Variable SI on DWI b_1000_ trace and highly variable ADC values	Chordoma, pituitary fossa meningioma
Sinonasal papilloma	Soft tissue density mass often with bony resorption and remodelling	Medium SI	High SI with typical “cerebriform” pattern (alternating lines of high and low SI)	Heterogeneous CE with typical “cerebriform pattern”	Non-specific pattern; intralesional carcinoma foci show lower ADC values than surrounding papillomatous tissue	Mucocele, sphenochoanal polyp, sphenoid sinus malignancies
Chordoma	Expansive soft tissue density lesion with intratumoural calcifications causing extensive lytic bone destruction	Medium–low SI	High SI. Foci of calcification show low SI	Moderate to marked heterogeneous CE. Sometimes “honeycomb” pattern	Variable, usually restricted diffusion, with the lowest ADC values found in dedifferentiated subtype	Ecchordosis physaliphora, arrested pneumatization, pituitary adenoma, chondrosarcoma
Nasopharyngeal carcinoma	Expansive soft tissue density mass extending from the nasopharyngeal area to the sphenoid bone	Low SI	A little higher SI than muscle	Lower CE than normal mucosa	Restricted diffusion	Osteomyelitis and several large masses with involvement of sphenoid and nasopharyngeal areas, such as metastasis, lymphoma, and adenoid-cystic carcinoma
Neuroendocrine tumour	Homogeneous isodense or mild hyperdense mass with bony destruction	Low- intermediate SI	Low-intermediate SI	Moderate and homogeneous CE. The “pigeon pattern” is often visible	Restricted diffusion	Other tumours involving paranasal sinuses with no very high SI on T2W images and sphenoid sinus inverted papilloma
Lymphoma	High density soft tissue mass with lytic destruction or bony remodelling of sinus walls	Intermediate SI	Mildly high SI	Moderate and homogeneous CE	Restricted diffusion with very low ADC values (typically < 0.6 × 10^−3^ mm^2^/s)	Nasopharyngeal carcinoma, neuroendocrine carcinoma, adenoid-cystic carcinoma, adenocarcinoma, metastases, sinonasal melanoma
Multiple myeloma/plasmacytoma	Usually multiple and punched-out lytic bone lesions. Plasmacytoma presents as a single lytic bone lesion without sclerotic borders	Focal myeloma lesions show low SI (hypointense to normal fatty marrow) with different patterns	Focal myeloma lesions show high SI with different patterns	Homogeneous CE	High SI on DWI and higher ADC values than normal bone marrow	Osteosarcoma, chondrosarcoma, malignant fibrous histiocytoma, bone Langerhans cell histiocytosis, lymphoma
Metastasis	Area of lytic bone destruction (except for osteoblastic lesions from prostatic cancer)	Low SI. High SI if from melanoma	Variable. Usually high SI, but also low or medium SI are common presentations	Variable CE, usually marked	Depending on DWI pattern of primary tumour, generally restricted diffusion	Primary malignant neoplasms, in particular chordoma, chondrosarcoma and plasmacytoma

## Developmental lesions (Table 1)

### Ecchordosis physaliphora

**Fig. 1:**
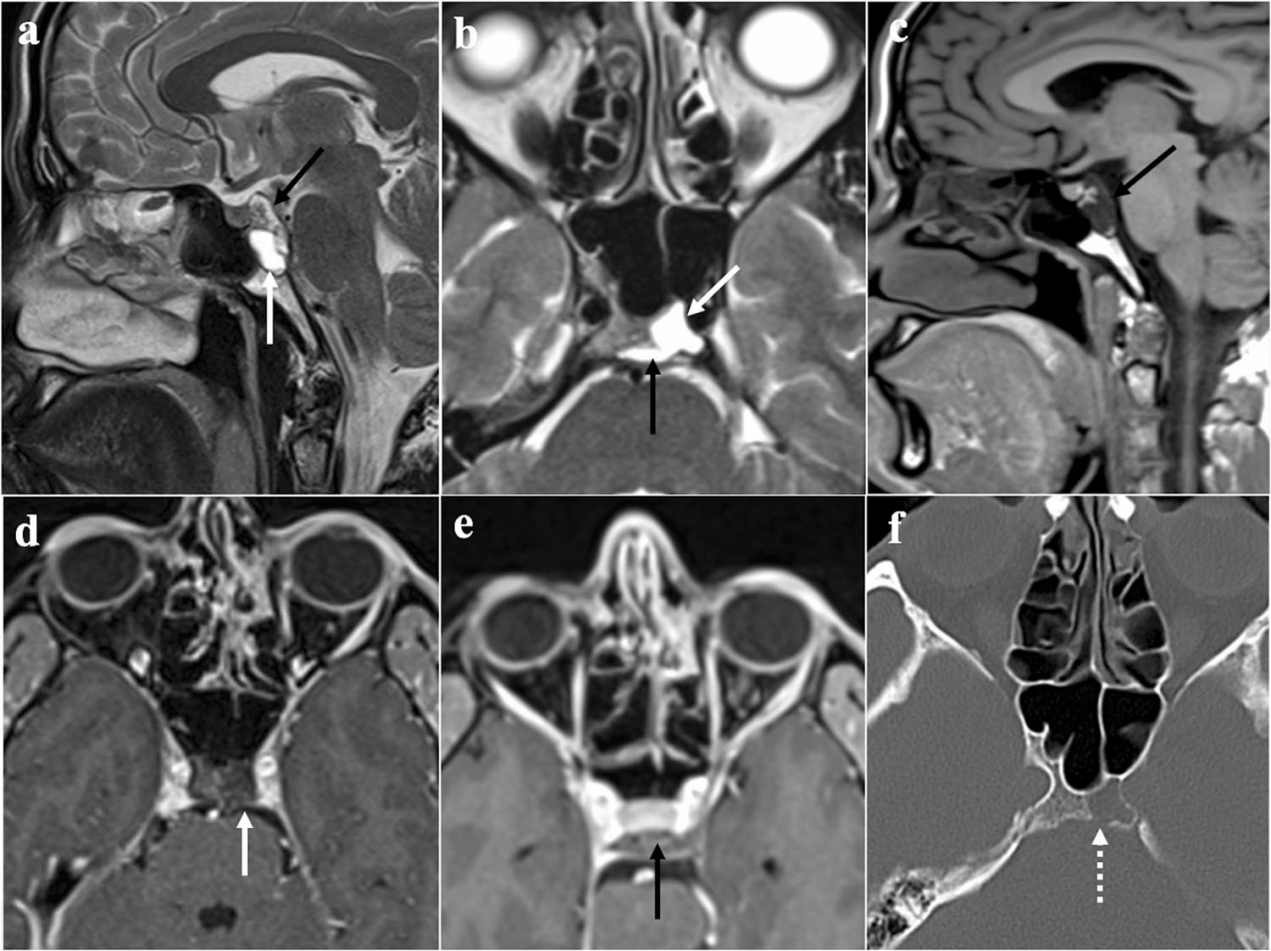
Ecchordosis physaliphora in a 24-year-old male patient with headache. MRI shows a midline, intradural, cystic lesion located in the retroclival prepontine region (black arrows) with intraosseous extension into the dorsal aspect of the clivus (white arrows). It shows T2 high (**a**, sagittal; **b**, axial), T1 low SI (**c**, sagittal), and lack of enhancement after gadolinium contrast media intravenous injection (**d**, **e**, axial). CT (**f**) reveals a bony defect in the dorsal clivus representing the stalk (white dotted arrow) connecting the retroclival and intraosseous components of the lesion. Note the well-marginated and scalloped bone margins of the lesion in the dorsal clivus

Ecchordosis physaliphora is a midline congenital gelatinous haemartoma not bigger than 2 cm, derived from notochordal remnants (Fig. 1). It is usually located in the intradural space of the prepontine cistern at the same level as the Dorello’s canal, and it is attached to the dorsal wall of the clivus by a small peduncle, the typical “stalk sign” [13]. CT features include a well-defined bony clival defect with cortical preservation and the bony (or cartilaginous) “stalk” projecting from the clivus. MRI shows an expansive lesion with low signal intensity (SI) on T1W images, high SI on T2W images, lack of contrast enhancement (CE), and facilitated diffusion on diffusion-weighted imaging (DWI) [19].

*Differential diagnoses*: Bony erosion — albeit this feature is reported to be extremely rare for notochordal remnants — joint with a restricted diffusion, should raise the suspicion of clival chordoma, which is considered to be the malignant counterpart of ecchordosis and its main mimicker [13]. Other differential diagnoses include dermoid cyst, a mass of the midline with no CE but high SI on T1W, and arachnoid cyst that can be distinguished because it presents the same SI as cerebrospinal fluid on all sequences [20].

### Neurenteric cyst

**Fig. 2:**
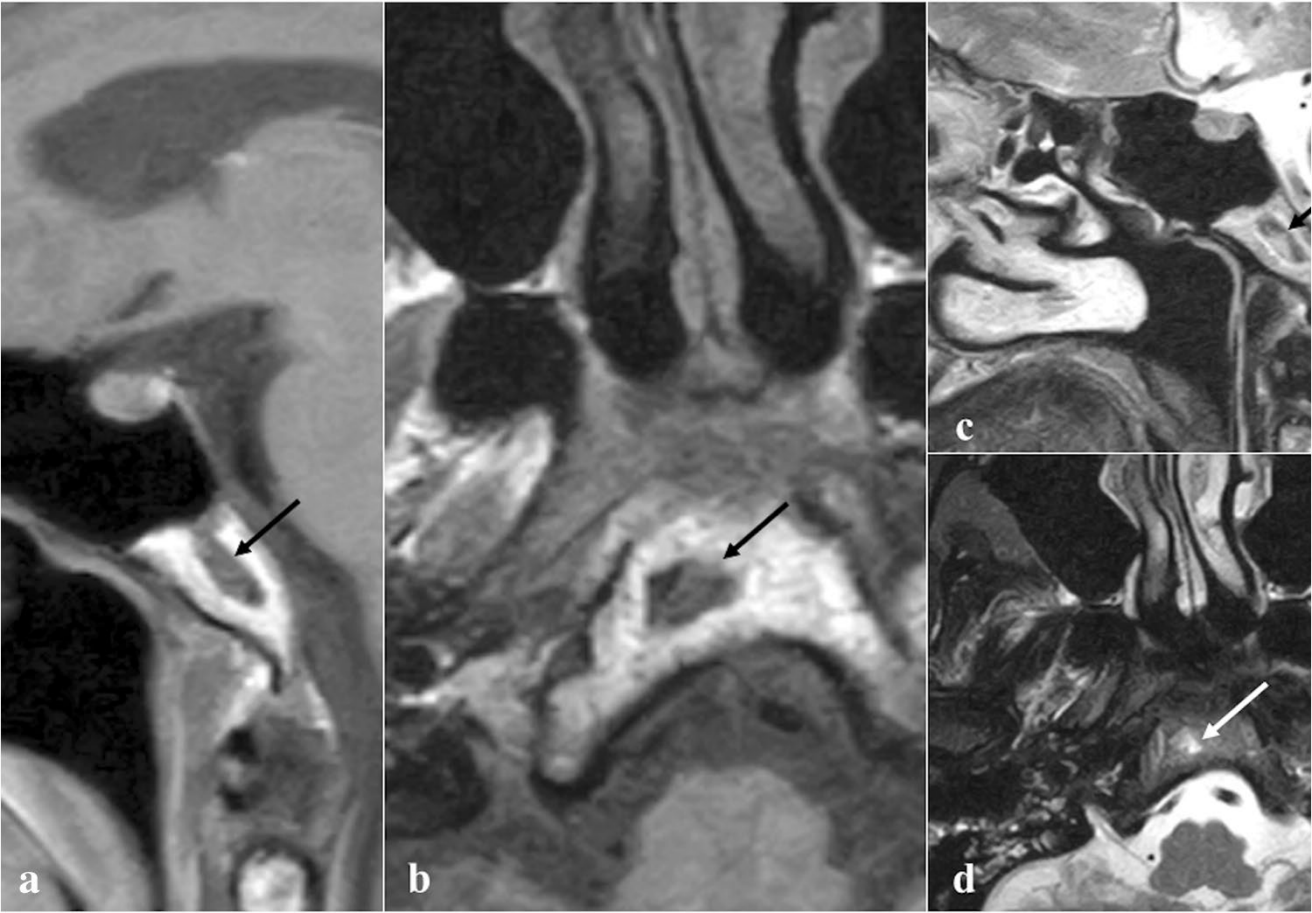
Neurenteric cyst of the clivus as an incidental finding in a 56-year-old female patient with headache. MRI shows an oval, intramedullary cystic lesion of the clivus (arrows). Compared to the cerebrospinal fluid, this lesion is characterised by intermediate-to-high SI on sagittal (**a**) and axial (**b**) T1W images, and high SI on sagittal (**c**) and axial (**d**) T2W images, thus reflecting high protein content

Neurenteric cyst develops from the incomplete resorption of the canal of Kovalevsky (neurenteric canal), the embryological connection between the neural tube and foregut (Fig. 2). It is lined by mucin-secreting intestinal-type or respiratory epithelium [21]. CT density and MRI T1W SI are quite variable, but they are more often high because of the proteinaceous material within the cyst [21]. High SI on T2W images and facilitated diffusion on DWI are also generally observed [22]. Internal septa, enhanced walls, and bony alterations are not common.

*Differential diagnoses*: dermoid and the rare proteinaceous epidermoid cyst (“white epidermoids”), which show high SI on T1W as neurenteric cyst but moderate to striking diffusion restriction; arachnoid cyst, which follows cerebrospinal fluid SI on all sequences; nerve sheath tumours can be easily distinguished because they show a strong CE, and they are very unlikely to be located in the midline because of their association with cranial nerves [23].

### Arrested pneumatisation of the sphenoid sinus

**Fig. 3:**
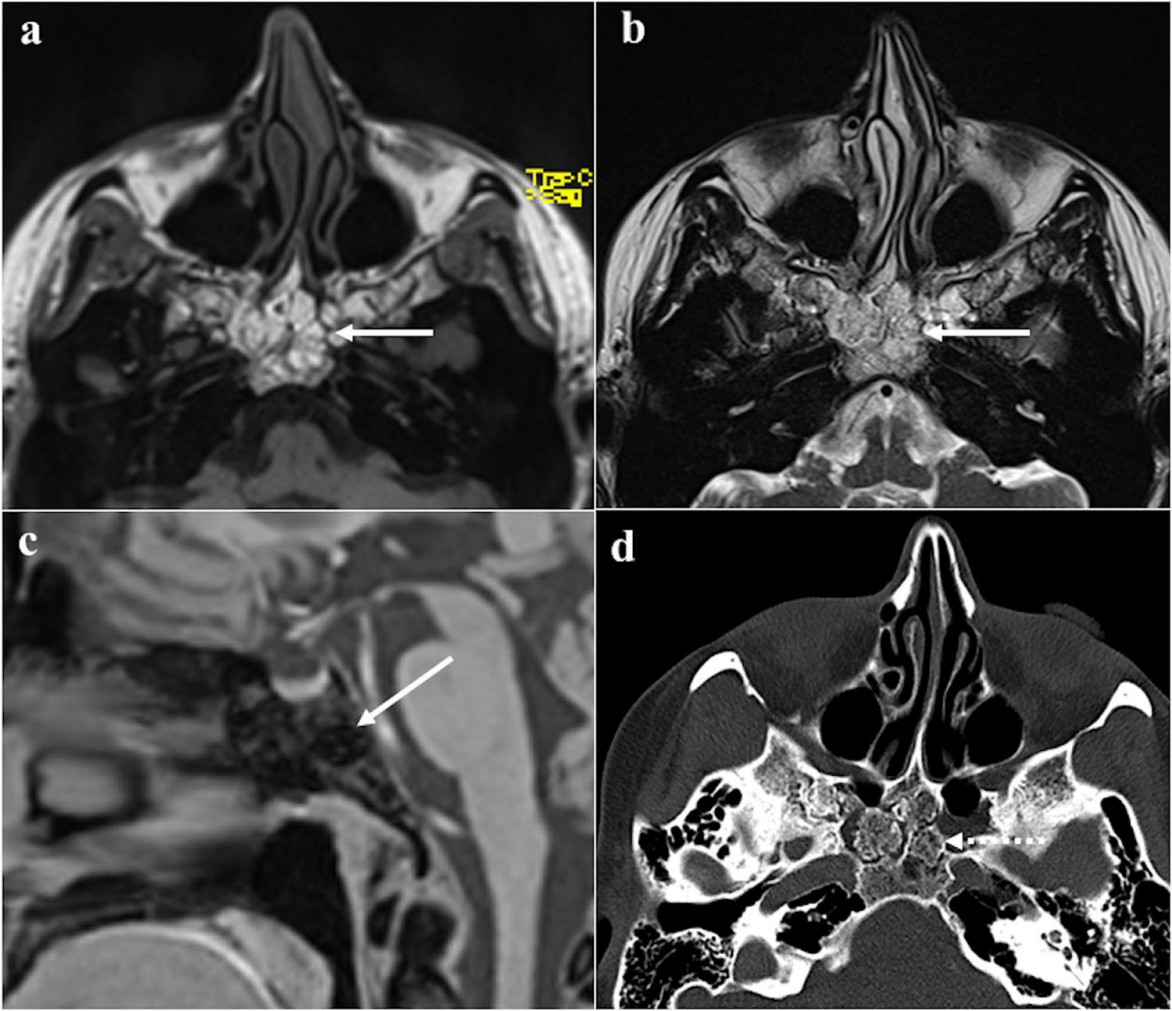
Arrested pneumatisation of the sphenoid sinus as an incidental finding in a 51-year-old female patient with headache. The sphenoid sinus is replaced by a non-expansile solid lesion (white arrows) showing high SI on MRI axial T1W (**a**) and T2W images (**b**), and homogeneous low SI on sagittal T1W fat-saturated sequence (**c**). Axial bone algorithm reconstruction CT image (**d**) shows a lesion with sclerotic margins, internal curvilinear calcifications, foci of fat, and loss of bone trabeculae (white dotted arrow). Note the absence of a cortical bone breach

Arrested pneumatisation of the sphenoid sinus is basically a variant of skull base development (Fig. 3) which can be found in up to 7.4% of the population [24]. Although often it represents an incidental finding, it can sometimes be associated with nonspecific symptoms, mostly headache [25]. Physiologically, the sphenoid sinus is absent at birth, and its body is completely formed by the red bone marrow. From the second to the fourth month of life, a process of yellow bone marrow conversion and subsequent respiratory epithelium colonisation begins, and it ends only with adulthood [26, 27]. Incomplete pneumatisation occurs whenever, for unknown reasons, such a process is interrupted, and yellow marrow foci persist in adults. This phenomenon may actually involve other paranasal sinuses, such as the ethmoid [28]. Its non-expansive nature, the presence of osteosclerotic margins, linear calcifications in the matrix, and the absence of cortical breaches and of intralesional foci of fat on MRI representing yellow bone marrow are all very specific signs [29].

*Differential diagnoses*: fibrous dysplasia, which shows a “ground-glass” bone marrow appearance on CT and an expansile nature with possible involvement of neural foramina [29]; ossifying fibroma**,** which can be distinguished thanks to its a more expansile nature and because its matrix more closely resembles to the ground-glass pattern of fibrous dysplasia [29]; chordoma and chondrosarcoma tend to be expansile and destructive in nature, without central fat [29]; osteomyelitis and metastases usually show a variable pattern of bony destruction and a low T1 SI of bone marrow [29]; intraosseous lipoma shows instead a fatty matrix with microcalcifications and a variable degree of trabecular bone loss with associated cortical breach [30]; intraosseous haemangioma has a typical “sunburst appearance” and CE on CT, an expansile nature, and it does not necessarily contain fat, while it may reveal cortical breach [30].

### Epidermoid cyst

**Fig. 4:**
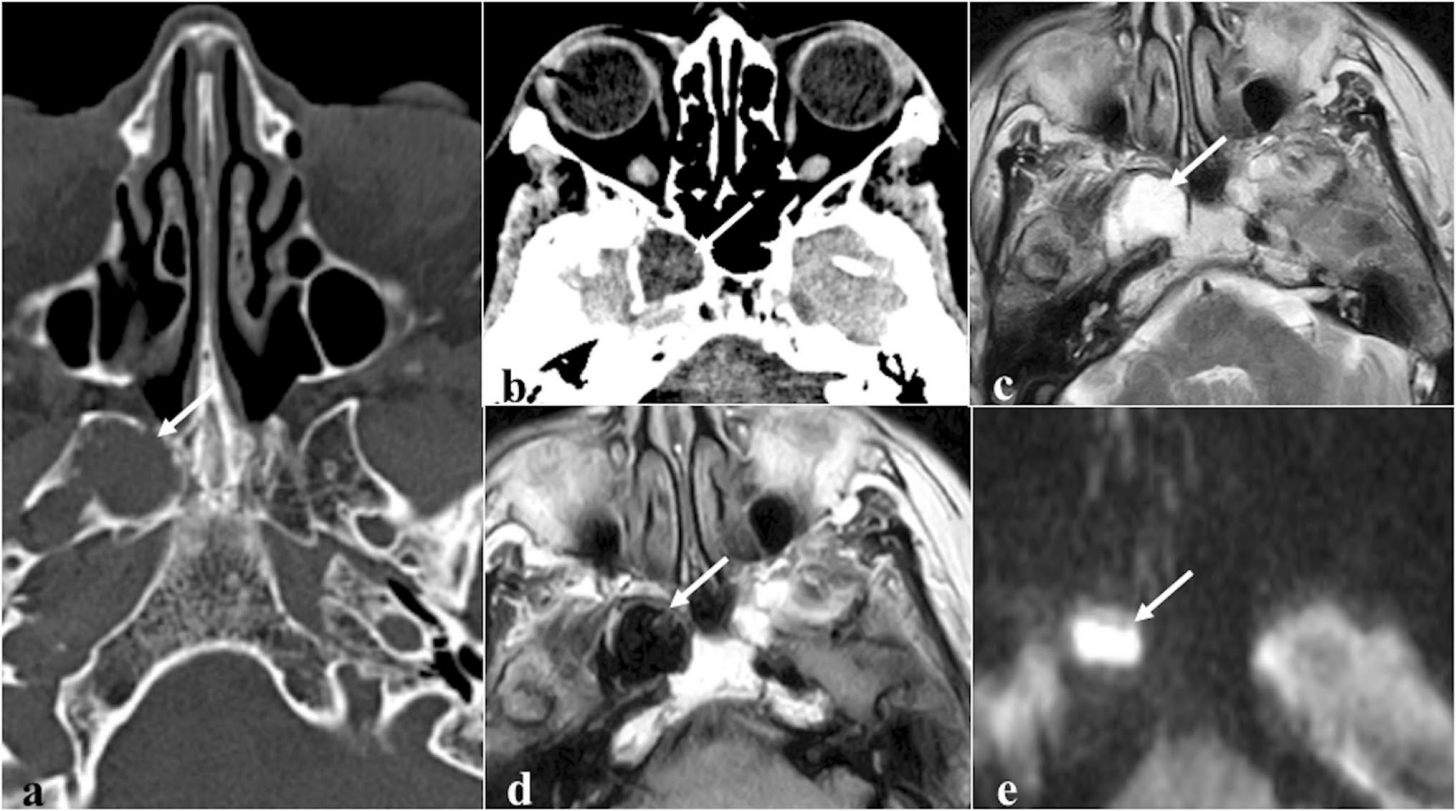
Sphenoid epidermoid cyst as an incidental finding in a 74-year-old female patient. Axial CT images (**a** and **b**) reveal a rounded lytic lesion in the right greater sphenoid wing (white arrows) with sclerotic margins and homogenous density similar to cerebrospinal fluid. Axial MRI images show that the lesion has high SI on T2W (**c**), heterogeneously low/dirty SI on fluid attenuated inversion recovery (**d**), and high SI on DWI b1000 sequences (**e**) due to the restricted water movements. Epidermoid cyst has similar features as arachnoid cyst on CT. Arachnoid cysts would have demonstrated homogeneous low SI on fluid attenuated inversion recovery MRI — as low as cerebrospinal fluid — and facilitated diffusion on DWI

Epidermoid cyst is a rare, slow-growing, benign lesion of ectodermal origin lined by squamous epithelium, and containing laminated keratin (Fig. 4). It differs from dermoid cysts for the lack of cutaneous adnexa or mesodermal elements such as hairs [31]. The cerebellopontine angle and suprasellar area are the most often involved sites. Paranasal sinus localization is exceedingly rare with only a few case reports [32]. Since sphenoidal epidermoid cysts are frequently located into the diploe, they are thought to originate mainly from congenital entrapments of ectodermal elements within the presphenoid fusion plates [32]. At CT, epidermoid cysts appear hypodense with a lack of CE or, sometimes, with a thin peripheral rim of enhancement. A scalloped or lobulated sclerotic rim is typical [33]. Epidermoid cysts appear similar to cerebrospinal fluid on MRI T1W (low SI) and T2W (high SI) images. High SI on T1W images and high density on CT may be rarely observed due to protein deposits (“white epidermoids”) [32]. Heterogeneous suppression of SI on fluid-attenuated inversion recovery (FLAIR) sequence, high SI on DWI, and low apparent diffusion coefficient (ADC) values — due to the intralesional squamous epithelium and keratinaceous debris that hinder the free water diffusion — are typical [34].

*Differential diagnoses*: arachnoid cysts, which follow cerebrospinal fluid SI without restricted diffusion [31]; abscess, which differentiates from epidermoid cyst for a ring CE with thicker walls and a surrounding oedema [35]; dermoid cysts, neurenteric cysts, or mucoceles are instead difficult to differentiate from a “white epidermoid” even though their clinical management does not differ in practice [32].

### Fibrous dysplasia

**Fig. 5:**
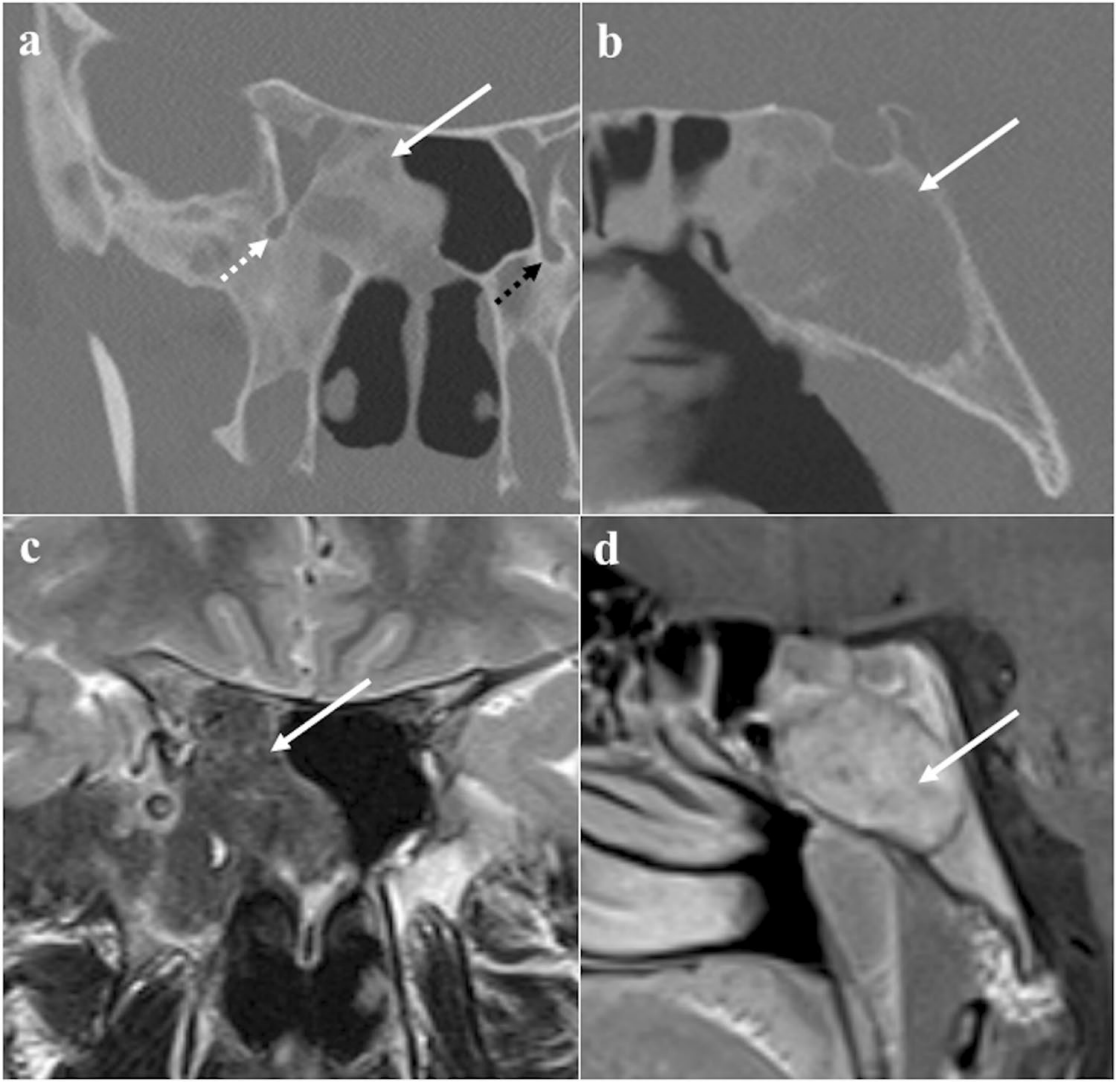
Fibrous dysplasia in a 20-year-old male patient with right atypical trigeminal neuralgia. Coronal CT image (**a**) reveals an expansile lesion in the middle cranial fossa extending into the right sphenoid sinus, pterygoid plates, sphenoid wings, and parietal bone with a “ground glass” appearance representing fibrous tissue (white arrow). Notice the narrowing of the right foramen rotundum (white dotted arrow) compared to the contralateral (black dotted arrow). Sagittal CT image (**b**) shows expansion of the clivus (arrow). At MRI, the lesion shows low SI on coronal T1W image (**c**, arrow) and highly inhomogeneous enhancement on sagittal T1W CE image (**d**, arrow)

Fibrous dysplasia is a congenital disease that is characterised by an altered osteoblastic differentiation resulting in the replacement of normal bone with poorly organised and structurally unhealthy fibrous tissues (Fig. 5) [36]. Monostotic (single bone) or polyostotic (multiple bones) forms are recognised. Fibrous dysplasia may be isolated or part of systemic conditions, especially “McCune-Albright syndrome” (fibrous dysplasia, café-au-lait skin spots, and precocious puberty) and “Mazabraud syndrome” (fibrous dysplasia plus intramuscular myxomas) [36]. Malignant transformation — most commonly into an osteosarcoma — occurs in up to 2.5% of cases [37]. CT is considered the diagnostic gold standard, and bony alterations can show three different patterns: ground-glass (56%), homogeneously dense (23%), and cystic (21%). The attenuation coefficient values typically vary from 70 to 130 Hounsfield unit [37]. Instead, MRI is less accurate, and it shows variable T1-T2 SI, depending on the content of the alterations: active lesions with metabolically active fibrous tissue show intermediate T1 SI and high T2 SI, whereas inactive lesions with highly mineralized matrix have low T1 and T2 SI [38]. Active lesions also show high SI on CE-T1W images, while DWI does not exhibit water diffusivity restriction [39].

*Differential diagnoses:* MRI can be very misleading, and especially when fibrous dysplasia alterations show T1 intermediate SI, T2 high SI, and vivid CE, it could resemble a skull base malignancy. In this circumstance, it is very useful to perform a CT that will solve any doubt by revealing the typical fibrous dysplasia bony changes [38]; Paget disease is a chronic, idiopathic progressive condition characterised by initial bone destruction followed by reparative processes. The sphenoidal involvement has been anecdotally reported in this condition, but the fact that other sinuses are usually spared, the rare and scarce cyst-like changes and the occurrence after the fifth decade are all useful features for differentiation from fibrous dysplasia [40]. Intraosseous meningioma — compared with fibrous dysplasia — shows feathering of lesions edges, soft tissue involvement and vivid homogenous CE [41]. Ossifying fibroma is a benign tumour with similar features, but it typically shows better-defined boundaries with osteosclerotic shells, whereas the periphery of fibrous dysplasia usually blends with the surrounding bone [42]. Malignant transformation of fibrous dysplasia into osteosarcoma should be suspected by the presence of periosteal response on CT and by areas of restricted diffusion on MRI, although these are not specific signs [39, 43].

## Inflammatory lesions (Table 1)

### Fungus ball (mycetoma)

**Fig. 6:**
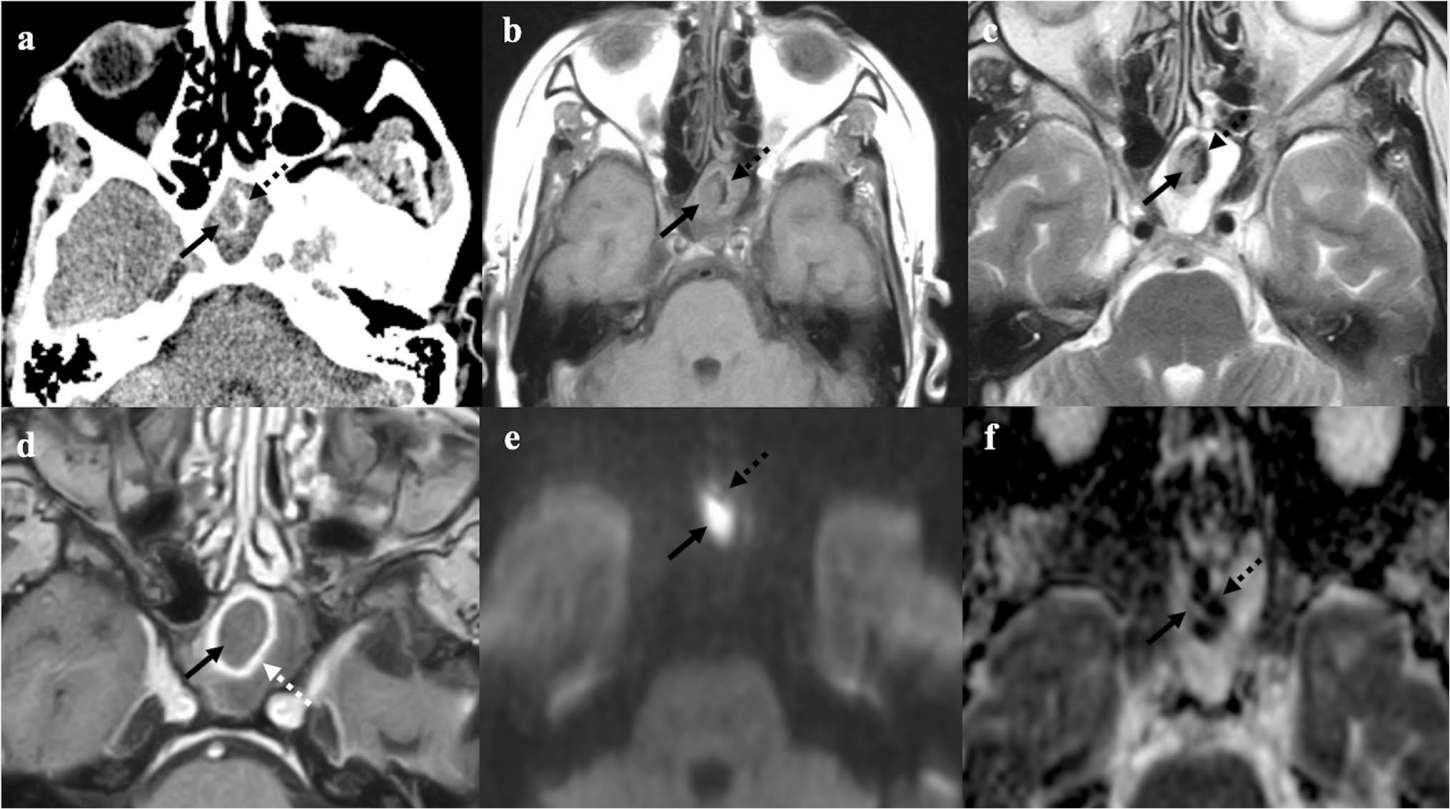
Fungus ball of the left sphenoid sinus (black arrows) in a 61-year-old female patient complaining of headache. CT shows a soft tissue density mass within the left sphenoid sinus with peripheral foci of calcific deposit due to fungal hyphae (black dotted arrows). Complete sinus opacification indicates obstruction of the ipsilateral spheno-ethmoidal drainage recess (**a**). MRI shows a mass in a completely mucous-filled left sphenoid sinus: the lesion is characterised by intermediate-to-low T1W (**b**) and T2W (**c**) SI and intralesional calcified foci with very low SI (black dotted arrows) similar to the air signal. Peripheral rim enhancement is seen on the axial T1W image obtained after gadolinium contrast injection (white arrow, **d**). The fungus ball shows intralesional areas of low SI on b800 DWI trace (**e**), and very low ADC values (**f**) due to the presence of calcifications and paramagnetic metals of fungal hyphae (black dotted arrows). These findings are suggestive for non-invasive fungal infection

Fungus ball is the most common form of non-invasive fungal rhinosinusitis in immunocompetent non-atopic patients (Fig. 6). It is probably related to a deficient mucociliary clearance that fosters the intraluminal extramucosal fungal proliferation, and *Aspergillus fumigatus* is the most commonly causative organism [44]. Fungus ball appears as a hyperdense mass — due to the conglomerated fungal hyphae — with linear or punctuate central calcifications on CT. Mucosa is thickened and vividly enhanced after contrast agent administration, whereas the intraluminal content shows no CE. The sinus may be expanded with sclerotic bony walls and bowing deformity [44]. Fungus ball shows a typical very low or “dark” SI on T2W images because of both calcifications and densely packed hyphae containing paramagnetic materials (iron and manganese) that induce areas of signal void. SI of fungus balls on T1W images is usually high [45], but intermediate or low intensities may be found [46]. On the other hand, the thickened sinus mucosa shows homogeneously hyperintense SI on T2W and CE-T1W images [44]. Paramagnetic metals, calcifications, and the absence of free water are the bases for the hypointensity of fungus balls on both DWI b1000 and ADC map [45, 46].

*Differential diagnoses*: mucocele, which can show variable T1 SI depending on protein contents (from low to high), typically appears hyperintense on T2W images, and it does not contain calcifications and metallic-density materials; sinonasal mucosal melanoma, which shows high T1 SI due to haemoglobin and melanin derivatives, but also CE of the internal component [47].

### Mucocele

**Fig. 7:**
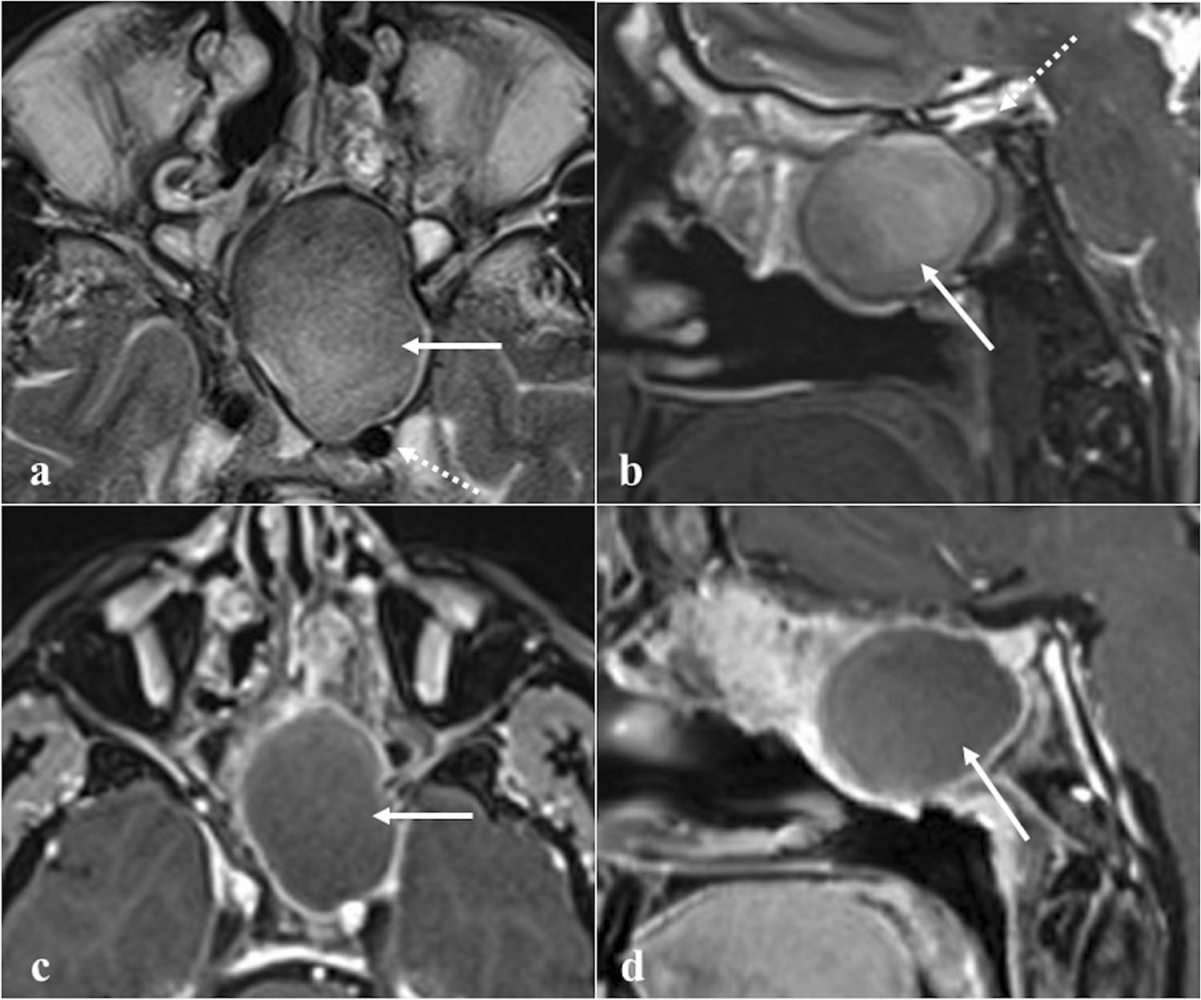
Left sphenoid sinus mucocele with high protein content in a 53-year-old male patient with headache. MRI shows a large mass (white arrows) displacing the ipsilateral internal carotid artery posteriorly (white dotted arrow) on T2W axial image (**a**) and the pituitary gland superiorly (white dotted arrow) on T2W sagittal image (**b**). Sphenoid sinus is markedly enlarged with mucous content and peripheral rim enhancement on axial (**c**) and sagittal (**d**) T1W fat-saturated CE images. No sign of superimposed infection or invasion of the adjacent structures is observed

Mucocele presents as a complete opacification of one or more paranasal sinuses: most frequently, the maxillary and ethmoid sinuses are filled with mucus and their walls are lined by normal-appearing respiratory epithelium (Fig. 7) [9]. It is often associated with bony wall expansion and focal osseous resorption. The pathogenesis of mucocele remains unclear, but an altered mucociliary clearance, cystic dilatation of the mucosal glands, and cystic development from embryonic remnants are all contributing factors, often in the context of chronic rhinosinusitis. Radiation therapy can also lead to an isolated sphenoid mucocele as a consequence of fibrous tissue formation obstructing the natural ostium [9]. Mucocele appears as a completely mucous-filled and expanded sinus with a typical rim enhancement — sign of encapsulation — and lack of intralesional CE. CT density and MRI SI are variable, depending on the amount of water, mucus, and proteins. Most frequently, this lesion exhibits high water content with low density on CT, low SI on T1W, and high on T2W images; the high protein content results in high CT density, high SI on T1W, and low SI on T2W images [9]. DWI and ADC are highly variable. When viscous protein-rich secretions are present, it may have very low ADC values (< 0.5 × 10^−3^ mm^2^/s) [48].

*Differential diagnoses*: fungus ball, which shows very low or "dark" SI on T2W [45]; sphenoid mucus retention cyst, a more common condition that shows similar SI and density but without completely filling the sinus, it does not determine bony changes nor it shows recognisable walls [49]; sphenoid sinus inverted papilloma, a rare entity that shows — contrary to the mucocele — homogeneous CE, also of the internal component [50].

### Osteomyelitis

**Fig. 8:**
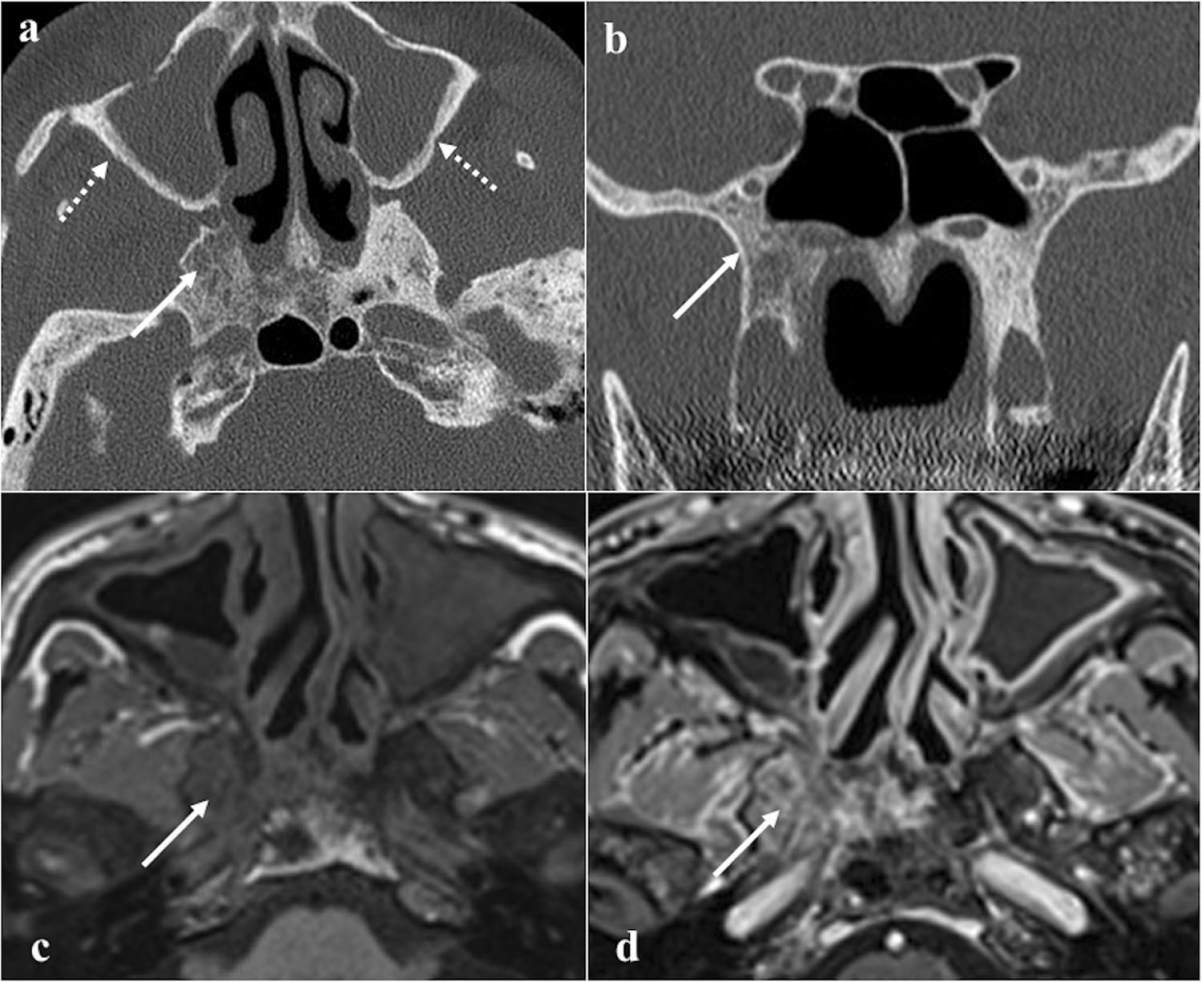
Sphenoid osteomyelitis in a 67-year-old male patient with chronic rhinosinusitis. Axial (**a**) and coronal (**b**) CT with bone algorithm reconstruction show maxillary sinusitis, osteolysis of the right greater sphenoid wing (white arrows) without cortical involvement, and thickening of maxillary sinus walls on both sides (dotted white arrows). Axial MRI images show inflammatory bony changes of the right greater sphenoid wing characterised by low SI on T1W image (**c**, white arrow) and mild enhancement after gadolinium contrast agent injection on T1W fat-saturated image (**d**, white arrow)

Skull base osteomyelitis including the sphenoid bone (Fig. 8) may arise without associated external malignant otitis or chronic suppurative otitis media, as opposed to the temporal bone involvement [10]. The identification of clival anomalies on imaging techniques is crucial to making a timely diagnosis [51]. Patients with sphenoid osteomyelitis often have predisposing conditions such as diabetes mellitus, longstanding corticosteroid use, HIV infection, and chronic rhinosinusitis [10]. Unlike temporal bone infections, many organisms other than *Pseudomonas aeruginosa* may be involved from transnasal swabs [52]. CT shows diffuse bone erosion and demineralization; after the administration of contrast medium diffuses soft tissue swelling, obliteration of fat planes, involvement of the skull base foramina, and vascular complications (thrombosis, compression, pseudoaneurysm) can be highlighted [53]. Focal or diffuse clival hypointensity on T1W images due to the replacement of the normal fatty marrow is the most consistent MRI finding. XII cranial nerve palsy may manifest in case of infiltration of the hypoglossal canal [54]. In the worst cases, abnormal soft tissues in the cavernous sinus, narrowing/occlusion of the internal carotid artery, meningeal enhancement, intracranial extension, and involvement of lateral structures (parotid gland, temporomandibular joint) can also be observed [53]. This inflammatory tissue usually shows hyperintensity on DWI trace and low values on ADC map; however, several authors have observed that ADC values in osteomyelitis are higher than malignant neoplasms [53].

*Differential diagnoses:* imaging of skull base osteomyelitis is nonspecific, thus making difficult the differential diagnosis with malignancy [53]. Differential diagnosis includes nearly all sphenoid/clival malignancies, especially nasopharyngeal carcinoma. The imaging findings are very similar, with CE-CT and CE-MRI showing an enhancing mass associated with bone erosion and infiltration and restricted diffusion; therefore, biopsy is often essential for a proper diagnosis [53]. Goh et al. [55], however, have suggested some MRI features that are useful for differentiation: in detail, involvement of lateral structures, soft-tissue enhancement, oedema, and abscess formation are more typical of osteomyelitis. Metastases can have a similar appearance, but the medical history pinpoints to the correct diagnosis [53]. Non-tumoural conditions such as fibrous dysplasia and Paget disease can be easily differentiated for the absence of soft tissue involvement [53].

## Benign tumours (Table 2)

### Haemangioma

**Fig. 9:**
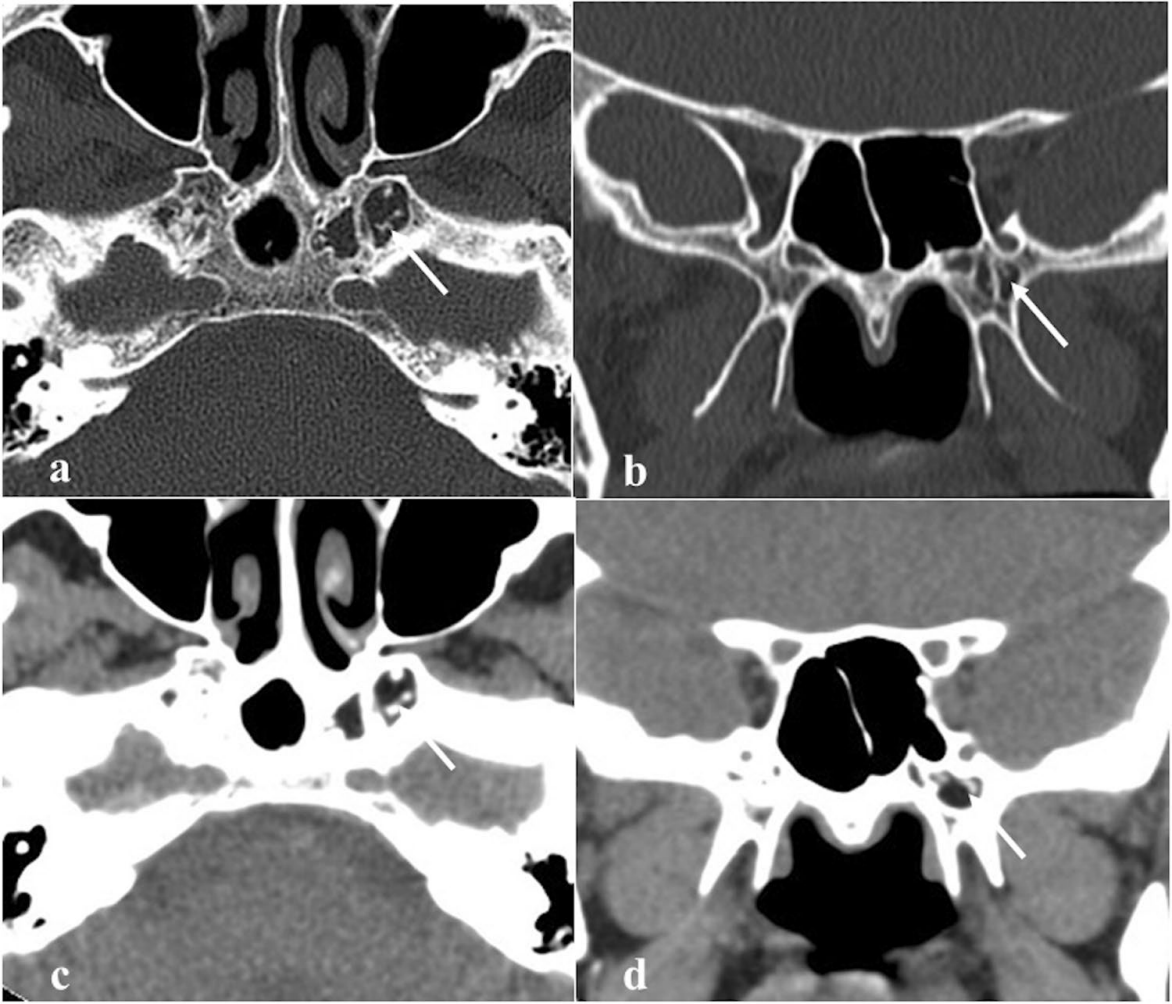
Sphenoid haemangioma as an incidental finding in a 70-year-old female patient with sarcoidosis and chronic rhinosinusitis. CT shows a small osteolytic lesion (white arrows) in the left greater sphenoid wing characterised by well-defined sclerotic margins and a “sunburst appearance” on axial (**a**) and coronal (**b**) images. The fatty component is found on axial (**c**) and coronal (**d**) soft tissue reconstruction algorithm images

Primary intraosseous haemangioma (Fig. 9) is a slow-growing haemartoma of blood vessels. CT shows an expansive, well-circumscribed area of osteolysis in the sphenoid bone with the typical “sunburst appearance” in which thickened trabeculae adjacent to abnormal vascular channels converge on a central area, with preservation of the periosteum [56]. On MRI, haemangioma usually appears “mottled” and heterogeneous with high SI both on T1W and T2W images due to the fat deposition and presence of slow-moving and pooled venous blood, respectively. However, haemangioma may show an atypical low SI on T1W and/or T2W images. Enhancement in CE-T1W images is usually observed because of its vascular channels [56]. DWI does not show restriction, with high SI on DWI trace and high ADC values, similar to the other skull haemangiomas [57].

*Differential diagnoses*: arrested pneumatisation of the sphenoid sinus, a fat-containing lesion that does not show the “sunburst appearance” (see above). In general, intraosseous haemangioma can be easily differentiated on CT from other benign osteolytic conditions — such as fibrous dysplasia or lipoma — due to the presence of the typical “sunburst appearance.” Absence of cortical aggressiveness differentiates sphenoid haemangioma from malignant osteolytic conditions such as multiple myeloma or histiocytosis. [58].

### Ossifying fibroma

**Fig. 10:**
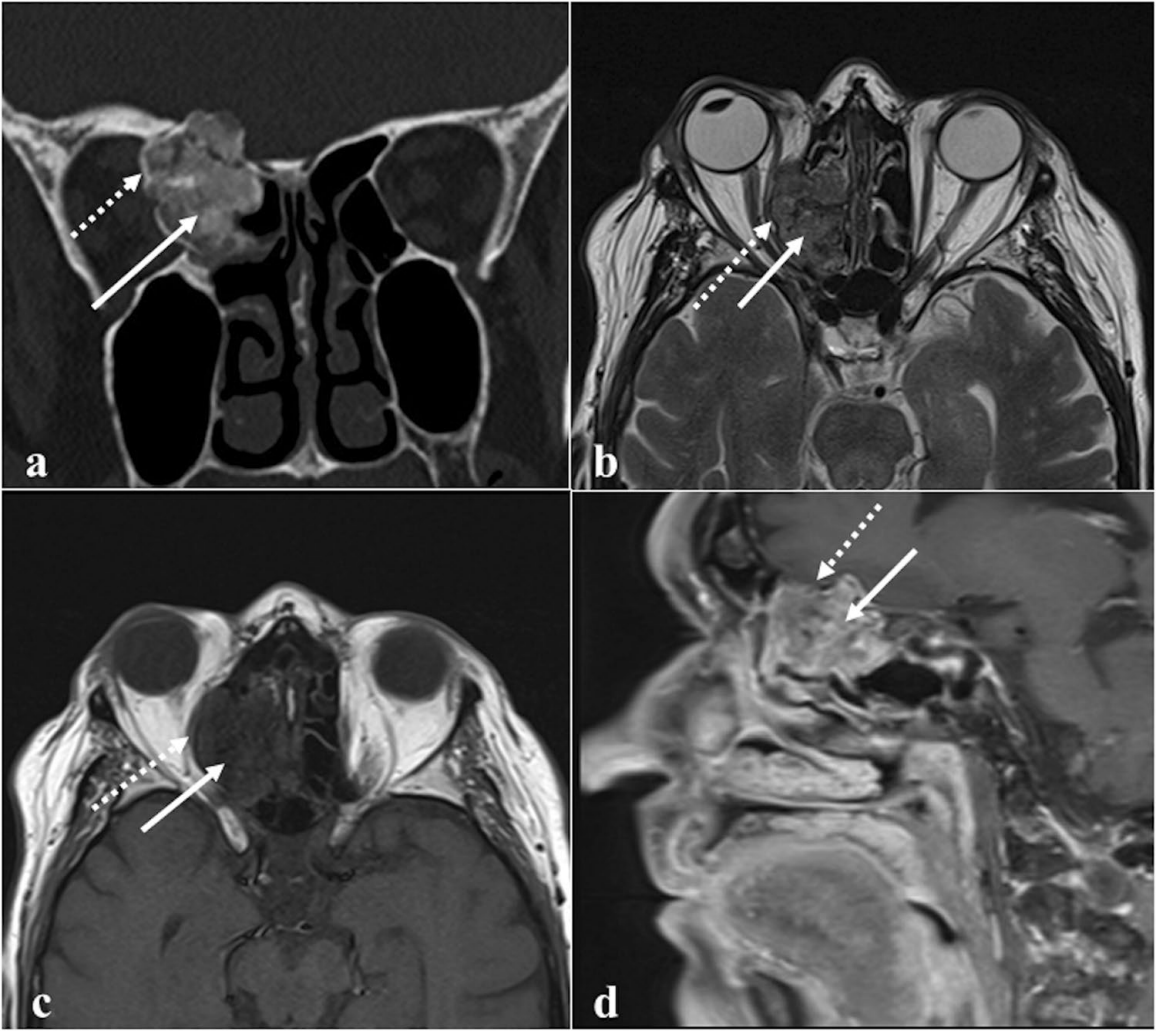
Right spheno-ethmoidal ossifying fibroma as an incidental finding in a 79-year-old female patient. Coronal CT image (**a**) shows a well-demarcated expansile lesion with central fibrous density areas (white arrow), surrounded by an ossified rim (white dotted arrow). MRI (**b**, **c**, and **d**) shows a lesion with intermediate central SI (fibrous areas, white arrows) and a peripheral rim of low SI (ossified area, white dotted arrows). The central fibrous areas have low SI on axial T1W image (**b**), mixed SI on axial T2W image (**c**), and inhomogeneous SI on sagittal T1W fat-saturated CE image (**d**). The peripheral ossified rim and internal septa appear hypointense in all MRI sequences

Ossifying fibroma is a benign fibro-osseous tumour characterised by the replacement of normal bone with dense fibrous tissue with foci of mineralisation (Fig. 10) [59]. It typically affects the mandibular bone, whereas sphenoid involvement is rare [59]. Ossifying fibroma is characterised by rapid growth, high recurrence rate (30–56%), a tendency to invade surrounding tissues — including the orbits — and bony erosions [60]. Imaging appearance varies according to the fibrous/bone tissue ratio within the lesion: in the early stages, a thick peripheral bony rim is observed which surrounds a fibrous soft tissue centre, whereas in the later stages, a progressive filling of this centre with mature bone can be detected [61]. CT depicts an expansive mass with heterogeneous CE, a sclerotic shell, and variable amounts of intralesional calcifications. Concomitant aneurysmal bone cysts with fluid–fluid levels are frequently identified [59]. MRI is important to assess the tumour extension, and it generally shows a lesion with two components: a central fibrous area with low T1 SI and mixed low–high T2 SI and a peripheral ossified rim with both T1 and T2 low SI. SI on T2W images is mixed high-low in case of cysts or more pronounced fibrous tissue. Ossifying fibroma reveals a heterogeneous moderate-high enhancement on CE-T1W images [62]. This lesion can also appear as a largely calcified mass completely hypointense on T2W [61].

*Differential diagnoses*: fibrous dysplasia, which shows ill-defined boundaries (see the above paragraph); arrested pneumatisation of the sphenoid sinus, which does not show an expansile nature (see before); osteoma, which — contrary to ossifying fibroma — does not show soft tissue CE, does not have the sclerotic shell, and usually does not exhibit adherence to soft tissues (dura mater) [63]; malignant tumours such as osteosarcoma or chondrosarcoma, which manifest ill-defined margins, contrary to the well and sharply defined shell of the ossifying fibroma [63]. When the ossifying fibroma is completely calcified, it can show both T1 and T2 low SI, an appearance similar to neoplasms with a high nuclear-cytoplasmic ratio, such as lymphoma and sinonasal melanoma**.** The differential diagnosis is facilitated by observing a vivid CE of these malignant entities and, on CT, the absence of intralesional calcium [61].

### Pituitary adenoma

**Fig. 11:**
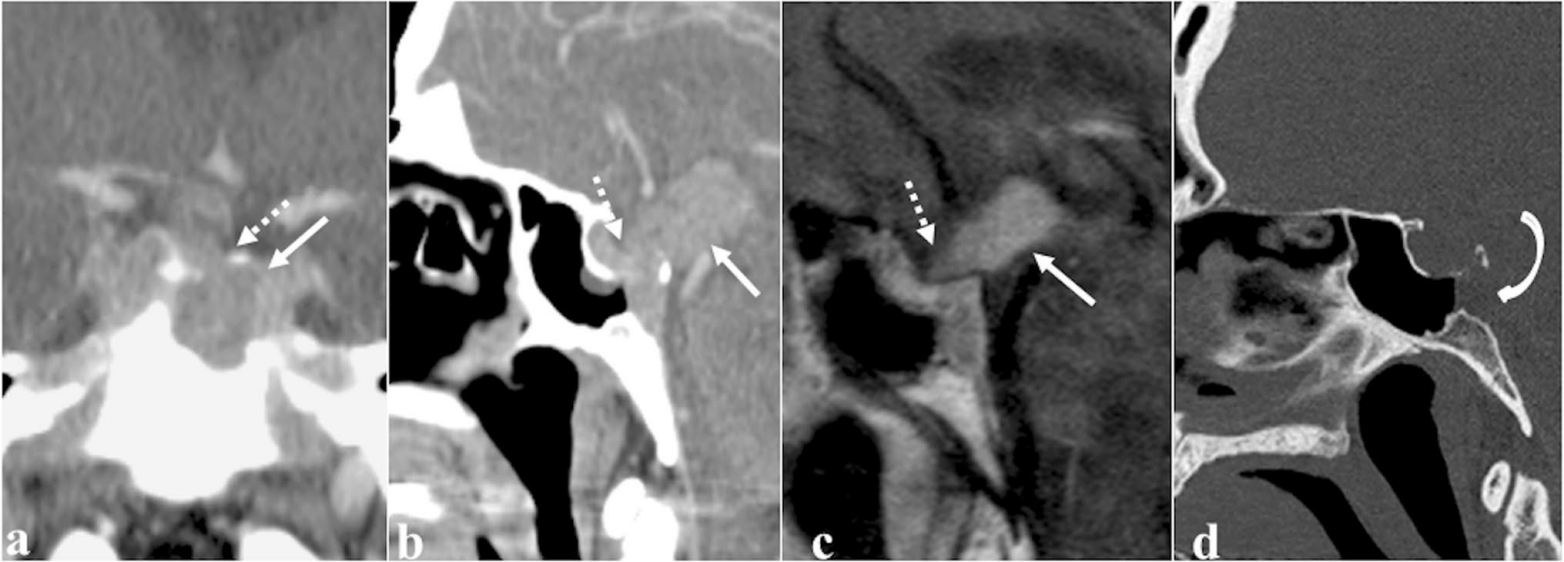
Invasive pituitary macroadenoma in a 60-year-old male patient with visual field defect. A huge pituitary macroadenoma (white arrows) extending into the suprasellar region through the pituitary stalk that invades the sella turcica and clivus. The “snowman” sign (white dotted arrows) is nicely depicted on coronal (**a**)—sagittal (**b**) CT sections and sagittal MRI T1W CE image (**c**) since the soft tumour is indented by the diaphragm sellae. This sign helps in differentiating macroadenomas from pituitary fossa meningiomas. Notice the focal erosion of the dorsal aspect of the clivus on the sagittal bone algorithm reconstruction CT image (**d**, white curved arrow)

Pituitary adenoma originates from the adenohypophysis and is classically divided by dimensions into micro- (< 10 mm) and macroadenoma (> 10 mm). The latter (Fig. 11) may cross the sellar floor and invade the skull base, especially into sphenoid and cavernous sinuses, clivus, orbits, dura mater, and subarachnoid, extradural, and nasopharyngeal spaces [64]. With their growth, they can show the typical “snowman sign” or “figure of 8”, given by the bilateral indentation of the macroadenoma by the diaphragma sellae [65]. Rarely, pituitary adenomas are entirely contained in the sphenoid body with the sellar floor intact since they originate from Rathke’s pouch ectopic remnants, trapped in the sphenoid ossification centres. Invasive macroadenoma appears as iso- or hypoattenuating compared to the brain tissue on CT, often with an inhomogeneous appearance due to cystic and/or haemorrhagic internal areas. These tumours are also associated with destructive bony involvement, well detectable on CT [66]. On MRI, macroadenoma presents medium–low SI on T1W images, medium-slightly high SI on T2W images, variable SI on DWI trace, and highly variable ADC values. CE is usually homogeneous [67].

*Differential diagnoses*: chordoma shows a higher T2 and DWI b1000 SI and higher ADC values than invasive macroadenoma, due to difference in histopathologic features (chordoma has an abundant myxoid stroma containing physaliferous cells with large cytoplasm, invasive macroadenoma shows highly crammed cells with large nuclei) [67]. Pituitary fossa meningioma could resemble a pituitary macroadenoma; nevertheless, two main aspects allow to distinguish these different conditions since meningioma reveals a more avid and homogeneous CE than adenoma [66], and in case of meningioma, the normal pituitary gland can still be recognised [65]. Moreover, meningioma does not show the “snowman sign” [65]. On the other hand, invasive macroadenoma may displace the cavernous segment of the internal carotid artery laterally, a non-detected pattern in meningiomas [68].

### Sinonasal (Schneiderian) papilloma

**Fig. 12:**
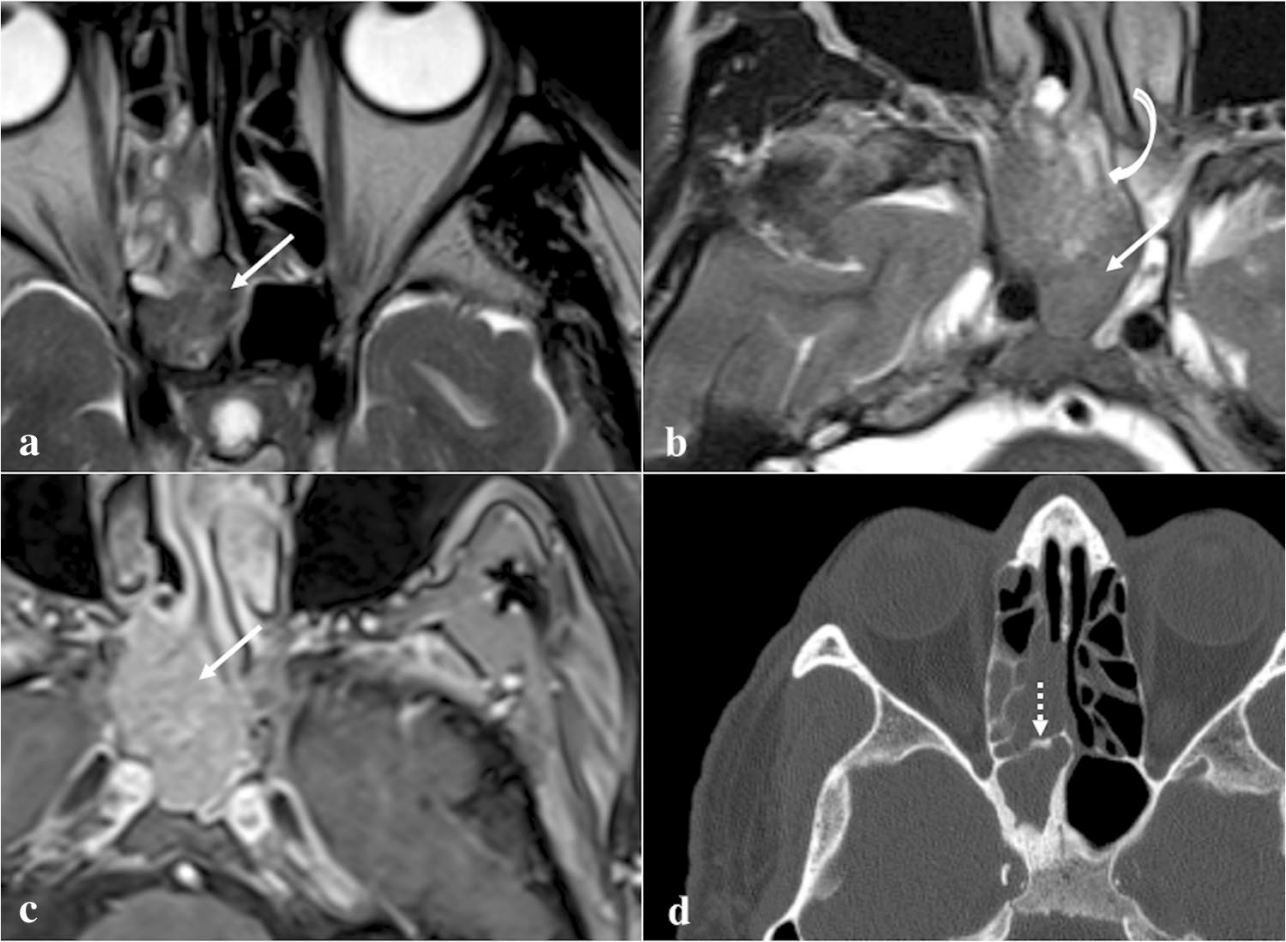
Inverted papilloma of the right sphenoid sinus in a 71-year-old male patient. MRI shows a solid expansive lesion in the right nasal fossa in correspondence to the spheno-ethmoidal recess (white arrows). That lesion has similar SI to the grey matter on axial T2W images (**a** and **b**) with focal “cerebroid” appearance (**b**, white curved arrow) and moderate enhancement on T1W fat-saturated CE image (**c**). Axial bone reconstruction algorithm CT reveals a focal plaque-like hyperostosis in the anterior wall of the right sphenoid sinus (**d**, white dotted arrow), as the likely site of tumour origin

Sinonasal papilloma is a benign tumour arising from the ectodermally derived Schneiderian membrane lining the nasal cavity and paranasal sinuses. Papillomas of the sphenoid sinuses are almost all of the inverted type (Fig. 12) [69, 70]. They show a typical endophytic growth pattern, invasive nature (70% of them cause bony erosion), high recurrence rates (20–50%), and a potential malignant transformation into squamous cell carcinoma (10%) [69, 71]. There is mounting evidence that some types of human papillomavirus represent a risk factor for malignant transformation [72]. Sinonasal papilloma presents as a soft tissue density mass which is often associated with bony resorption and remodelling (thinning and bowing) on CT. Focal hyperostosis (cone-shaped or plaque-like) into the sinus wall may indicate the site of tumour origin, and this must be surgically drilled so as to avoid recurrences. Calcifications within papilloma are rare. At MRI, this tumour appears isointense to muscle on T1W images [73], and it may show a typical “cerebriform” pattern on T2W and CE-T1W images, thanks to the alternating lines of high and low SI. The diffusion pattern is non-specific and does not reliably allow radiologists to separate the inverted papilloma from malignant lesions [73]; sometimes, intralesional carcinoma foci usually can be observed as areas of lower ADC values [74]. *Differential diagnoses*: sphenoid sinus mucocele, which does not show CE of the internal component (see “Mucocele” paragraph); spheno-choanal polyp, a unilateral inflammatory soft tissue mass arising from sphenoid sinus and extending through its drainage ostium and spheno-ethmoidal recess towards the choana and nasopharynx. The extension to the choana and the expansion of the sphenoid sinus ostium without bony erosions favour this latter diagnosis [75]; distinguishing an inverted papilloma from a sphenoid sinus malignancy on imaging is instead very complex. Despite high values on ADC map and typical “cerebriform” pattern are identified, an endoscopic biopsy is nonetheless necessary.

## Malignant tumours (Table 3)

### Chordoma

**Fig. 13:**
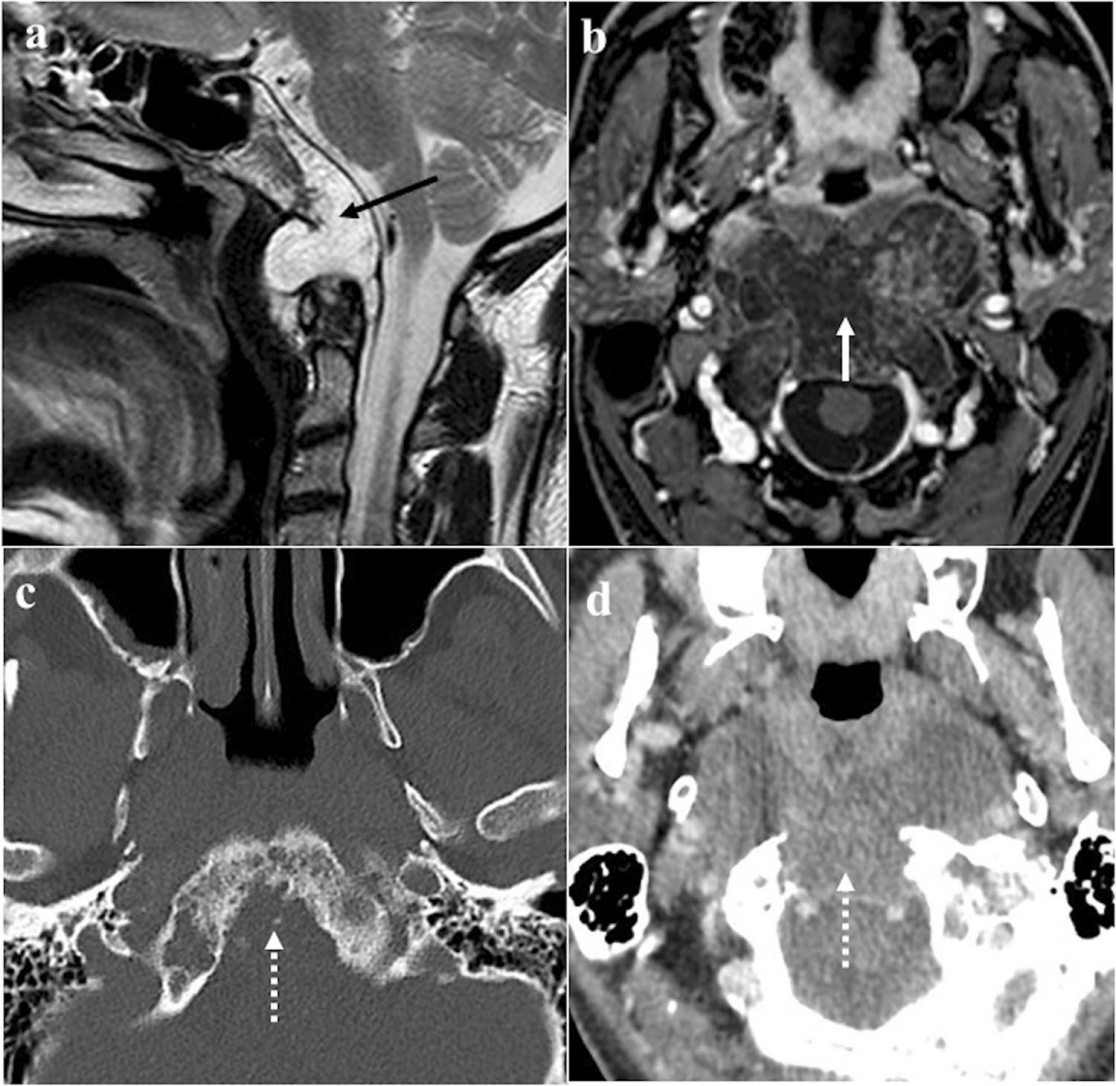
Chordoma of the clivus in a 41-year-old male patient with headache. The lesion appears as a destructive, multilobulated, well-circumscribed, expansile mass located in the midline next to the spheno-occipital synchondrosis. At MRI, high SI on sagittal T2W image due to the fluid content (**a**, black arrow) and honeycombing enhancement on axial T1W fat-saturated CE image (**b**, white arrow) are found. Axial CT images well depict a massive bony erosion of the clivus (**c** and **d**, white dotted arrows)

According to the 2021 WHO classification of central nervous system tumours, chordoma is classified as the only subtype of “notochordal tumours”, which belong to “chondro-osseous tumours” among other mesenchymal, non-meningothelial tumours [76]. It derives from undifferentiated, extradural remnants of the notochord, and four histological subtypes are recognised: conventional (most common, tumour cells embedded in hyaline cartilage-like stroma), chondroid (islands of cartilage formation), poorly differentiated, and dedifferentiated or sarcomatoid chordoma (chordoma associated with a high-grade sarcoma) [77] (Fig. 13). Intracranial chordoma accounts for around one-third of all chordomas [78]. It mostly arises from the clivus, whereas petrous apex, sella turcica, and sphenoid sinuses are often secondarily involved. Invasion of the prepontine cistern, foramen magnum, nasopharynx, chiasm, third ventricle, and jugular fossa are also common [79]. Clivus chordoma causes massive bony erosion, and it looks like a well-defined centrally located heterogeneous soft tissue density mass with hypodense foci of gelatinous degeneration on CT. Intralesional hyperdense foci are often found as calcified material — chondroid subtype — or sequestered/destroyed bone fragments [79]. Chordoma usually shows low-to-medium SI on MRI T1W images, and conventional chordoma shows typical features on T2W images, with high SI associated to hypointense intralesional septa which give a multilobulated appearance to the mass. Foci of calcification, blood, and mucus can be observed as well. Furthermore, dedifferentiated chordoma may show low SI on T2W images [78]. CE is very variable, and “honeycomb” appearance is frequently observed due to the presence of hypointense areas of necrosis and cartilage [79]. Regarding DWI, chordomas show a restricted diffusion, with the lowest ADC values found in dedifferentiated subtype (often < 1.0 × 10^−3^ mm^2^/s) [80]. However, DWI may primarily represent an important tool for differential diagnosis with chondrosarcoma (see below).

*Differential diagnoses*: ecchordosis physaliphora, which shows cortical preservation and higher ADC values (see “Ecchordosis physaliphora” paragraph); arrested pneumatisation of the sphenoid sinus, not expansile and with central fat; pituitary adenoma, which shows a lower T2 SI (see “Pituitary adenoma” paragraph); chondrosarcoma, a rare tumour which probably originates from malignant transformation of the cartilaginous cells of the synchondroses and that is composed of atypical chondrocytes with enlarged hyperchromatic nuclei set in an abundant cartilaginous matrix [81]. Features useful for differential diagnosis: chordoma is typically located more centrally, whereas chondrosarcoma arise laterally in the petro-clival synchondrosis; on CT, chondrosarcoma may show a typical “ring-and-arc” pattern of calcifications, due to endochondral mineralization of hyaline cartilage nodules; DWI reflects the differences in extracellular matrices of these entities: the cartilaginous stroma associated with variable grade of cellularity, typical of chondrosarcoma, is responsible of very high values in the ADC maps (often > 2.0 × 10^−3^ mm^2^/s), whereas chordomas usually show medium–low values [78].

### Nasopharyngeal carcinoma

**Fig. 14:**
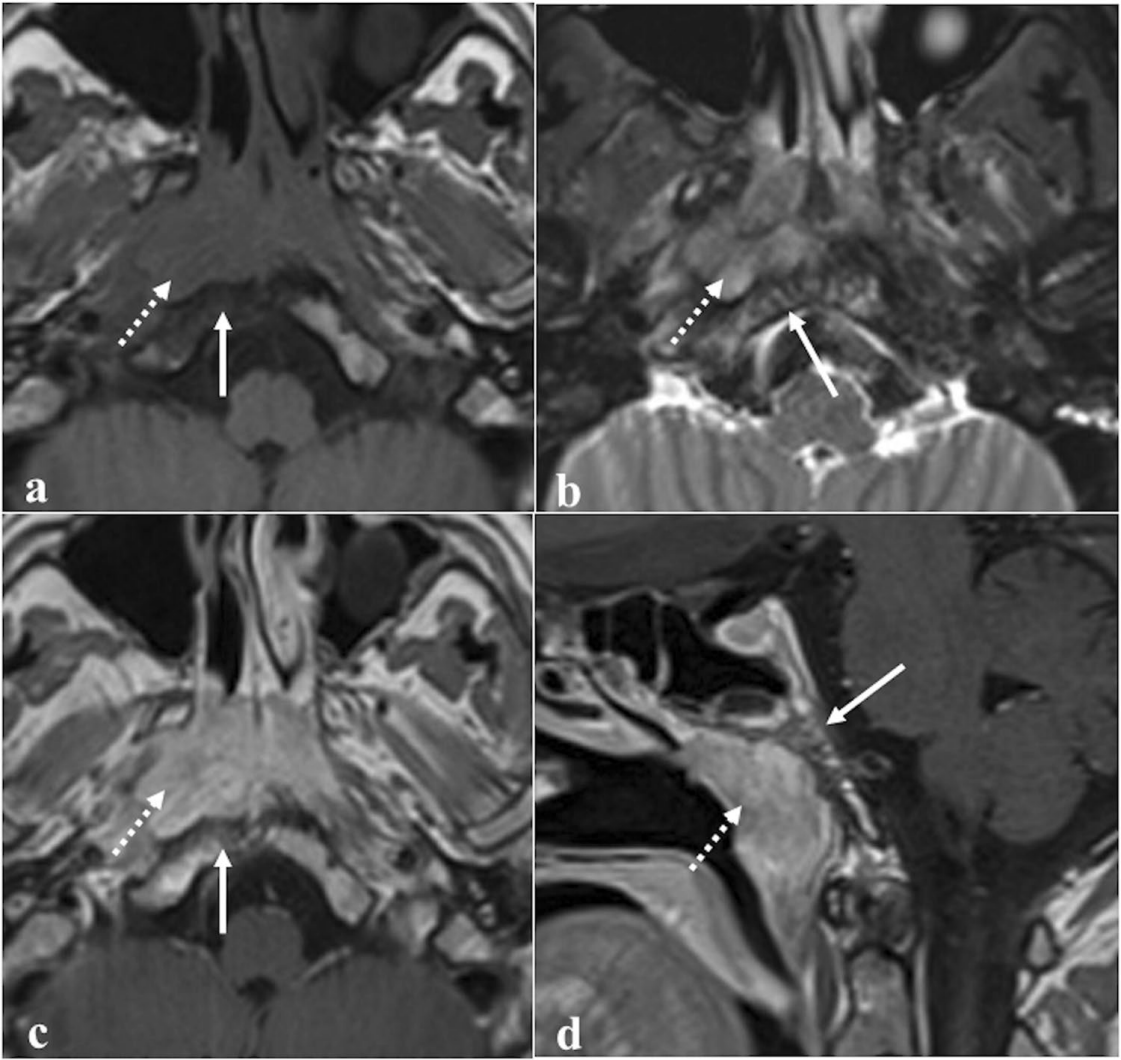
Nasopharyngeal carcinoma in a 28-year-old male patient. MRI shows a lesion of the right Rosenmüller fossa (white dotted arrows) invading the clivus posteriorly. Bony invasion (white arrows) is better depicted on axial T1W non-CE image as a focal area of low SI in the clivus next to the primary tumour (**a**). Bony involvement is less noticeable on axial T2W (**b**), axial (**c**), and sagittal (**d**) T1W CE images

Nasopharyngeal carcinoma normally originates from the Rosenmüller fossa (Fig. 14) and is histologically divided into three subtypes: keratinizing squamous cell carcinoma, nonkeratinizing squamous cell carcinoma, and undifferentiated or poorly differentiated carcinoma [82]. The keratinizing-type is related to tobacco, alcohol, and dietary nitrosamines exposure, whereas the other two subtypes are usually linked to Epstein-Barr virus infection. Nasopharyngeal carcinoma is more frequent in the Asian population (70%) [82]. Involvement of sphenoid sinus upstages the tumour to cT3; retropharyngeal/laterocervical metastatic lymph nodes are almost invariably detectable [83]. On CT, these tumours appear as a soft-tissue density mass with heterogeneous CE [83]. CT has high sensitivity in the detection of the sphenoid cortical bony erosion, whereas MRI is crucial to detect bone marrow invasion, well depicted on T1W images as a focal area of low SI [83, 84]. Nasopharyngeal carcinoma usually shows a slightly higher SI than muscle on T2W images, low SI on T1W images, a lower degree of CE compared to normal mucosa [84], and restricted diffusion [85, 86]. In the case of sphenoid invasion, it is fundamental to distinguish the frequent inflammatory reaction next to the neoplastic mass, by defining a precise inflammation-tumour border. Inflammatory changes usually show high SI intensity on T2W images, a thin superficial CE, and a facilitated diffusion on DWI, whereas neoplastic tissue shows lower SI on T2W images, a solid CE, a restricted diffusion [87]. Perineural invasion mainly through the trigeminal nerve may be observed.

*Differential diagnosis* is with other large masses involving the sphenoid and nasopharyngeal areas. Lymphoma often originates in the midline; frequently involves parotid and submandibular nodes, rarely the retropharyngeal nodes; and has lower ADC values than nasopharyngeal carcinoma because of its higher cellularity (usually ≤ 0.60 × 10^−3^ mm^2^/s) [84, 86]. Adenoid-cystic carcinoma arising from ectopic salivary tissue in the sphenoid sinus mucosa or from minor salivary glands of the nasopharyngeal submucosa may invade neighbouring structures including the clivus. It has a greater tendency compared to nasopharyngeal carcinoma to show perineural invasion through cranial nerves V and VII [5]. Metastases are suspected in case of a primary neoplasm elsewhere, and osteomyelitis is another possible lesion to be considered.

### Neuroendocrine tumour

**Fig. 15:**
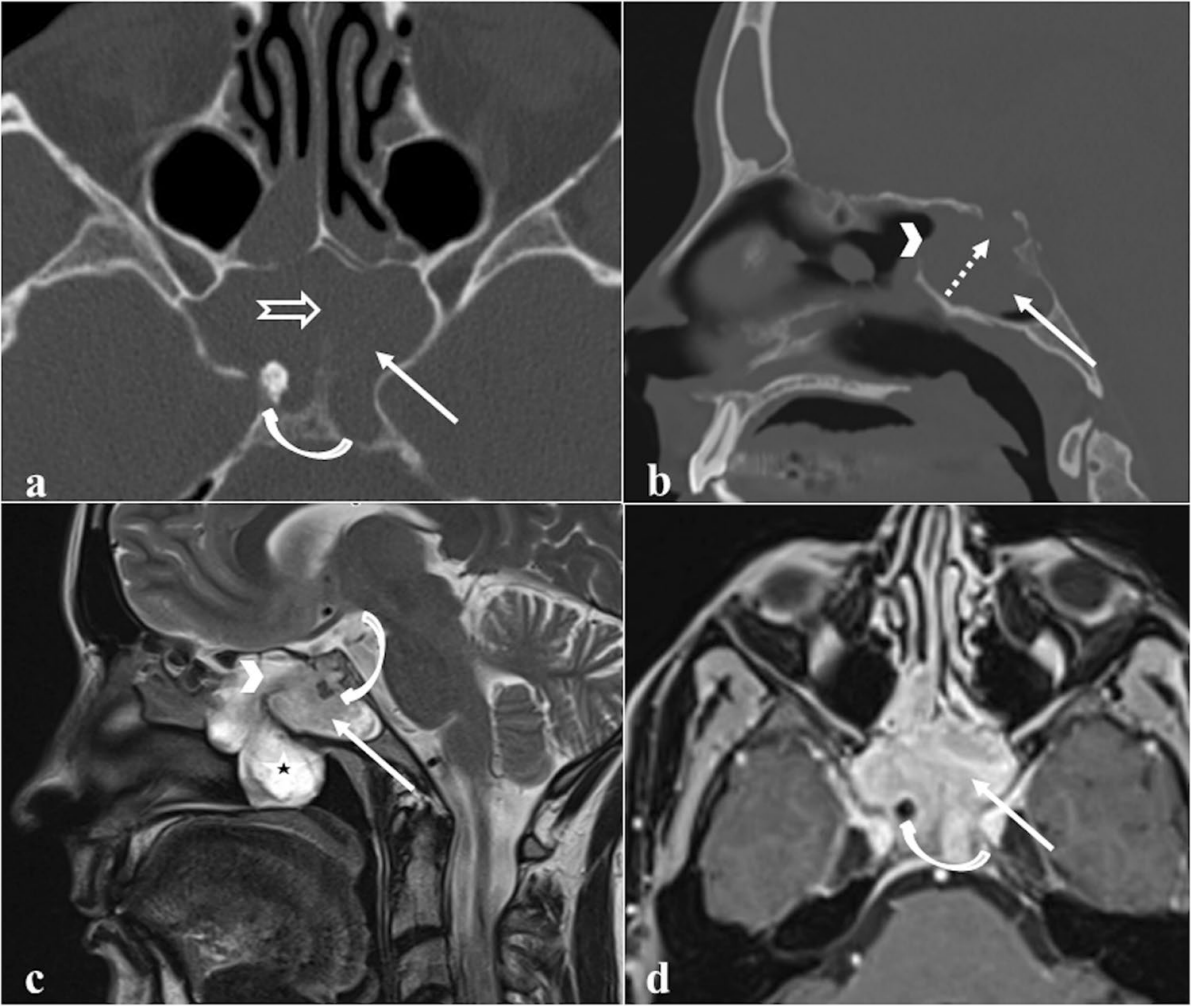
Neuroendocrine carcinoma of the sphenoid sinus in a 42-year-old female patient complaining of headache. CT with bone algorithm reconstruction shows massive opacification of both sphenoid sinuses (white arrows) with partial reabsorption of the intersphenoid septum on axial section (**a**, white empty arrow), and erosion of the floor of the sella turcica on sagittal section (**b**, white dotted arrow). MRI shows a solid mass replacing the right sphenoid sinus (white arrows) with low SI on T2W (**c**) and vivid enhancement on T1W fat-saturated CE images (**d**). Notice the right spheno-ethmoidal recess enlargement on sagittal section (**b** and **c**, white arrowheads) and the mucous retention in the right nasal fossa (**c**, *). An incidental osteoma in the right sphenoid sinus is found (**a**, **c**, and **d**, white curved arrows)

**Fig. 16:**
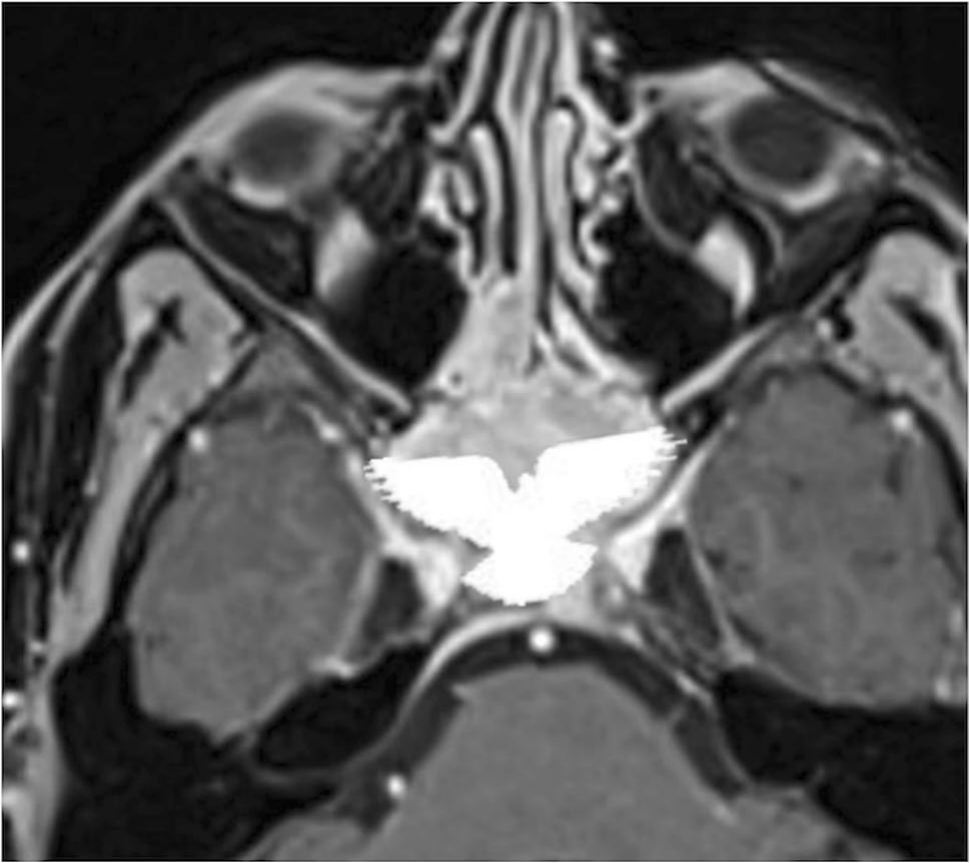
The so-called “pigeon pattern” of neuroendocrine carcinoma. Axial fat-suppressed T1-CE image with the schematic silhouette of a pigeon projected over the mass. Small cell neuroendocrine carcinoma with its growth can produce a symmetrical pattern, with a progressive and symmetrical invasion towards the head (anteriorly), the tail (posteriorly), and the wings (laterally) of the “pigeon”

Neuroendocrine tumour originates from neuroendocrine amine precursor uptake and decarboxylation cells (Fig. 15) [88]. It is categorised by cellular differentiation into well-differentiated (carcinoid), moderately differentiated (atypical carcinoid), and poorly differentiated (small cell neuroendocrine carcinoma) tumours [89]. The lower the degree of differentiation, the worse the patient’s prognosis. Paranasal sinus is an atypical location for a primitive neuroendocrine tumour, accounting for only 5% of cases. Small cell neuroendocrine carcinoma is the most frequent histotype found in the sphenoid sinus and the only one with sufficient CT-MRI data: it shows a homogeneous isodense or mild hyperdense appearance on CT given by the neuroendocrine “grana” characterised by closely packed cells. Calcifications and haemorrhage are very rare [90]. CT clearly shows the inevitably present bony destruction. Tumour growth usually shows a typical “pigeon” pattern with symmetrical involvement of the sphenoid, cavernous sinus, clivus, and internal carotid arteries (Fig. 16), and this is better detectable on T2W fat-suppressed images and T1W-CE images [90]. MRI shows low-intermediate SI on T1W and T2W images, with moderate and homogeneous CE [90]. DWI exhibits high SI in the trace with low ADC values [88].

*Differential diagnoses*: sphenoid sinus inverted papilloma, which shows a typically “cerebriform” pattern on MRI and may contain hyperdense foci on CT; sphenoid sinus squamous cell carcinoma, adenocarcinoma, and adenoid-cystic carcinoma are very difficult to distinguish only based on imaging because they often show bone erosions and intermediate SI on T2W, but also a more heterogeneous CE pattern with respect to neuroendocrine carcinoma; lymphoma exhibits similar SI on T1W and T2W and a similar homogenous CE (although not symmetrical or pigeon-like), but usually with lower ADC values than neuroendocrine tumour; olfactory neuroblastoma (esthesioneuroblastoma) can invade sphenoid bone, but it arises from the nasal cavity and shows peripheral areas of cystic degeneration and calcific foci [90].

### Lymphoma

**Fig. 17:**
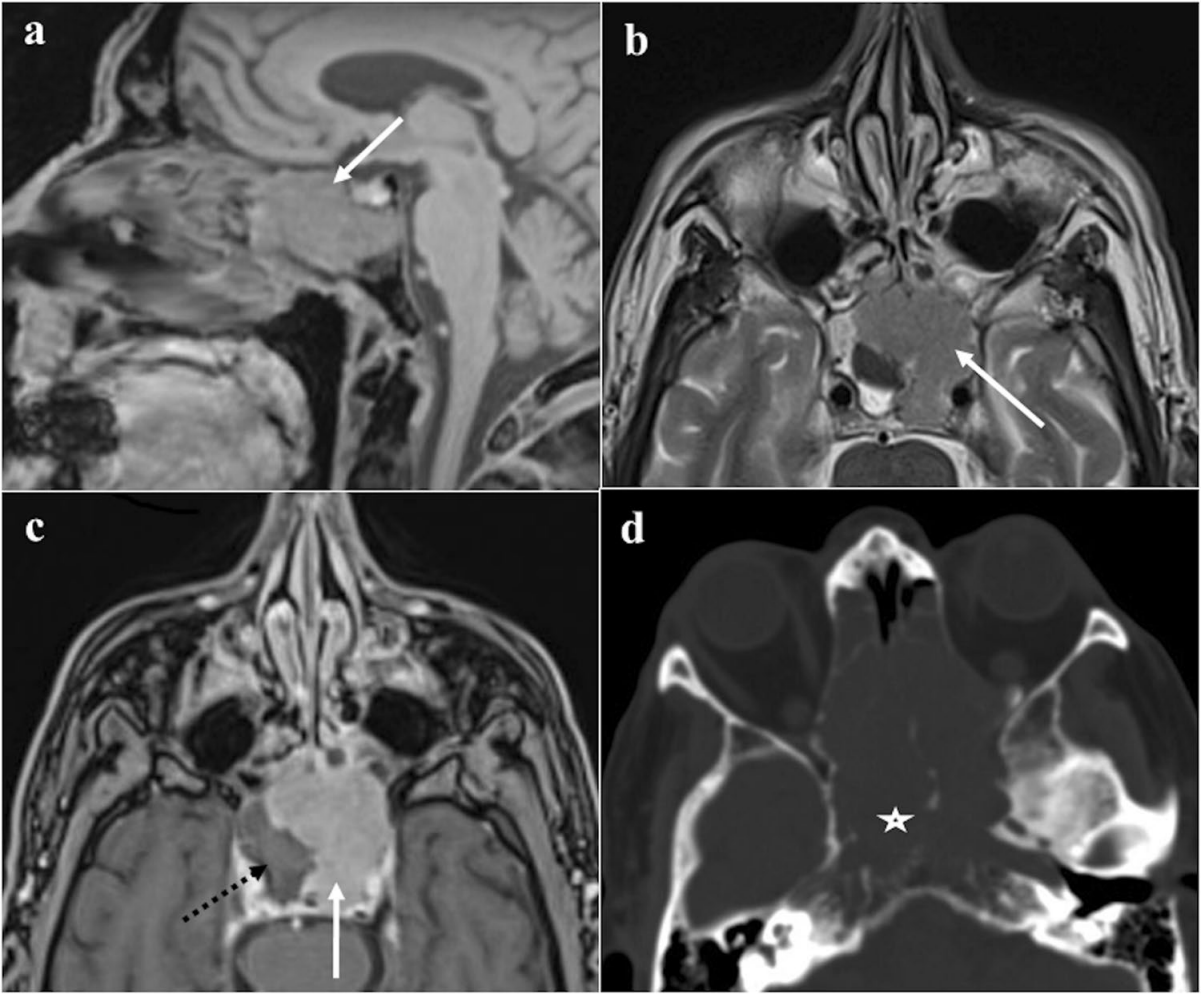
Primary lymphoblastic lymphoma of the left sphenoid sinus in a 79-year-male patient with headache. MRI reveals a homogeneous soft tissue mass in the left sphenoid sinus (white arrows) showing intermediate SI on sagittal T1W (**a**) and axial T2W (**b**) images and moderate homogeneous enhancement after intravenous gadolinium contrast agent on axial T1W fat-saturated images (**c**). The right sphenoid sinus is filled by partially dehydrated mucus due to the drainage obstruction (**c**, black dotted arrow). Bone algorithm reconstruction CT obtained one month later (**d**) shows a rapid growth of the lesion with massive destruction of the clivus (*)

Primitive lymphoma of the sphenoid sinus (Fig. 17) is exceedingly rare, with only 20 cases described in the literature [15]. Non-Hodgkin diffuse large B-cell lymphoma is the most common histotype. Perineural spread, bony destruction, and dura mater, cavernous sinus, and intracranial involvement is often observed [91]. Unenhanced CT shows as a soft tissue mass without calcifications, chondroid, or osteoid matrix. It usually has higher density than other neoplasms due to the high nuclear-cytoplasmic ratio. Intralesional haemorrhage and necrosis are rare. Permeative lytic destruction and bony remodelling of sinus walls are recurrent [92]. Isointense SI on T1W, mildly hyperintense SI on T2W, and moderate enhancement on CE-T1W images are generally described on MRI [86, 93]. Lymphoma shows a restriction of diffusivity with very low ADC values (typical values suggestive of lymphoma: ≤ 0.60 × 10^–3^ mm^2^/s) [85, 86, 94].

*Differential diagnoses*: all the sphenoid neoplasms characterised by bony erosions and intermediate SI both on T1W and T2W images: nasopharyngeal carcinoma, neuroendocrine carcinoma, adenoid-cystic carcinoma, adenocarcinoma, and metastases. Very low ADC values [95], homogenous CE, multiple lymph nodes echelons involved are typical — yet unspecific — features of lymphoma. Sinonasal melanoma usually shows a typical high SI on T1W for melanin and/or haemorrhagic foci [96, 97]. A completely calcified ossifying fibroma can show both T1 and T2 low SI, but it is easier to diagnose thanks to the absence of any CE. Moreover, the evidence of calcium on CT excludes the diagnosis of lymphoma [61].

### Multiple myeloma/plasmacytoma

**Fig. 18:**
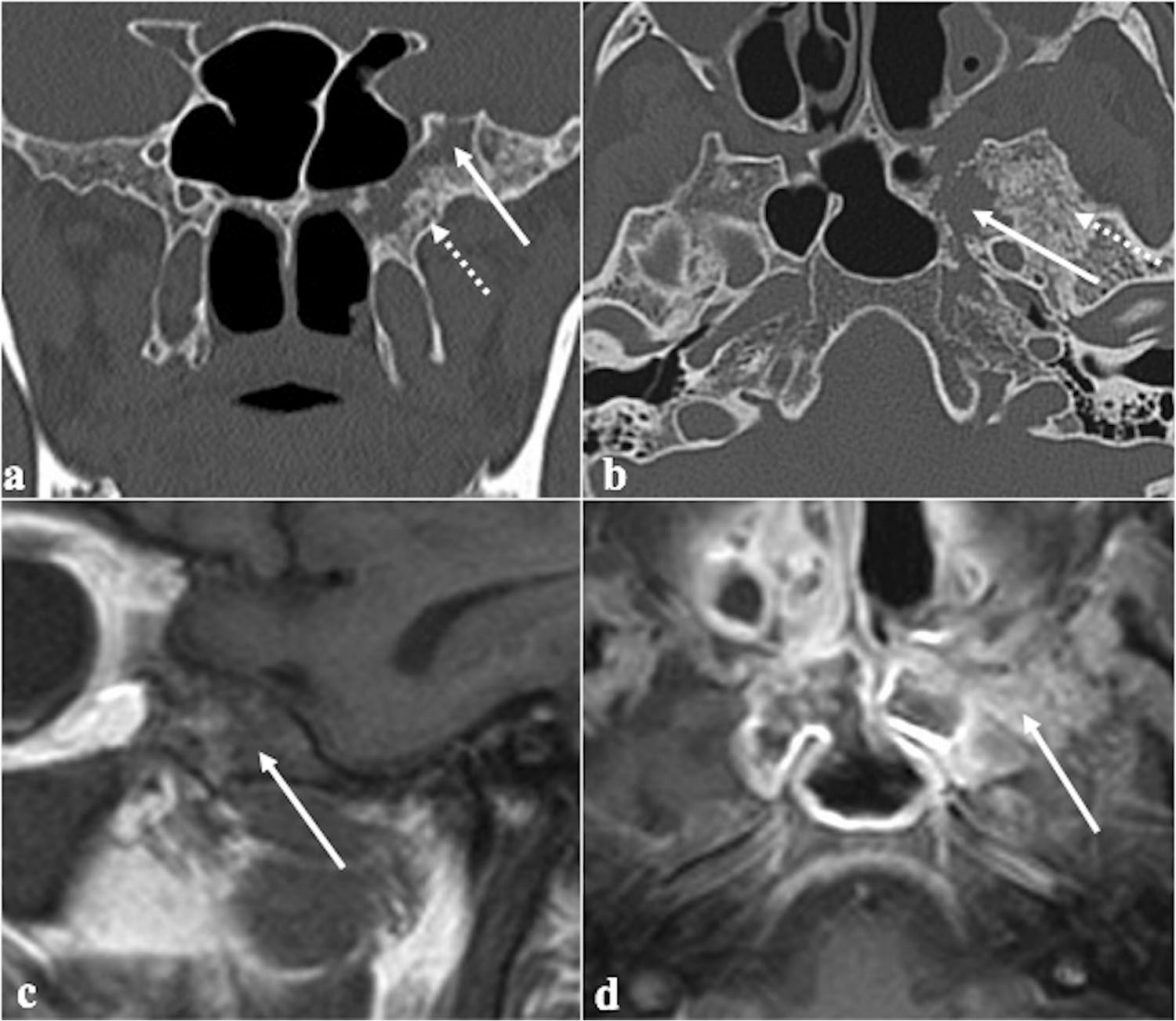
Sphenoid localization of multiple myeloma in an 82-year-old male patient. Coronal (**a**) and axial (**b**) CT images show a lytic lesion in the left greater sphenoid wing (white arrows) surrounded by bone sclerosis (white dotted arrows). MRI reveals a lesion in the left greater sphenoid wing characterised by low SI on sagittal T1W image (**c**, white arrow) and vivid enhancement after intravenous gadolinium contrast agent injection on axial T1W fat-saturated image (**d**, white arrow)

Multiple myeloma and plasmacytoma are both characterised by malignant proliferations of a single plasma cell clone (Fig. 18) [98]. Multiple myeloma bone involvement has four different patterns: disseminated form with multiple round lytic lesions (the most frequent subtype in the skull), a disseminated form with diffuse osteopenia, solitary plasmacytoma, and osteosclerosing fibroma [99]. In the former case, bone CT shows multiple lytic foci without a sclerotic rim, and that tend to coalesce (“punched-out” lesions); extramedullary extensions can be observed as expansive adjacent masses with soft tissue density. Plasmacytoma usually is represented by a single lytic bone lesion associated with a soft tissue density mass, but it is very rare at skull base level [98]. Multiple myeloma rarely manifests as multiple hyperdense osteosclerotic lesions (osteosclerosing form) [98]. Five different patterns are recognised on MRI: normal marrow (no visible infiltration); focal pattern; diffuse disease; salt-and-pepper appearance; and combined focal-diffuse infiltration. Multiple myeloma lesions show low SI on T1W images, mildly high SI on T2W images, and homogeneous CE. Myeloma infiltration presents high SI on DWI and higher ADC values than normal bone marrow in which very low free water diffusion is normally found [99].

*Differential diagnoses*: osteolytic malignant lesions (prevalent pattern in multiple myeloma) as osteosarcoma, chondrosarcoma, malignant fibrous histiocytoma, bone Langerhans cell histiocytosis, and lymphoma [100]. Differential diagnosis on imaging is difficult; “salt and pepper” pattern on MRI associated with “punched-out” osteolysis on CT are the most specific features.

### Metastases

**Fig. 19:**
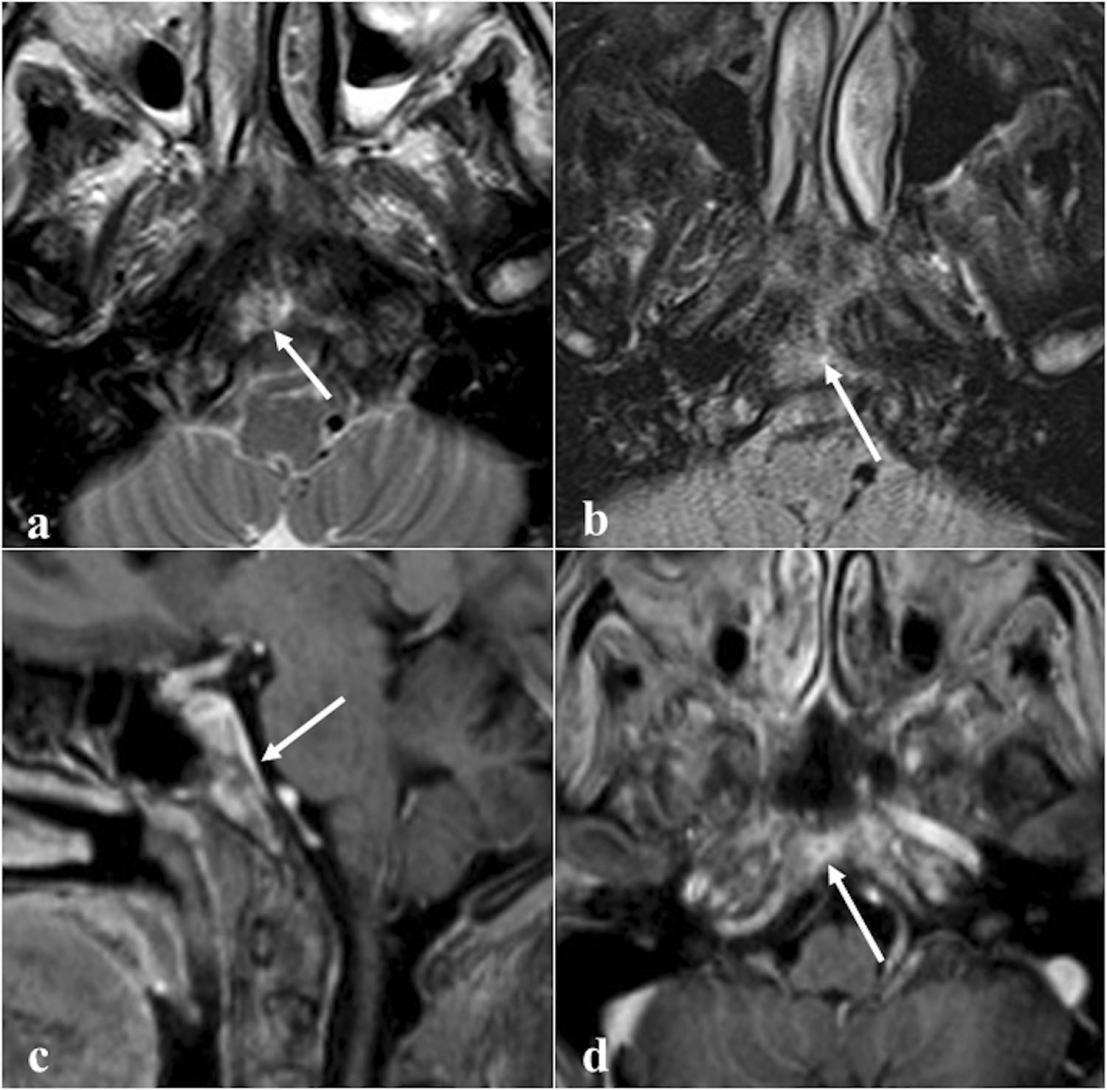
Clival metastasis in a 58-year-old female patient with known breast cancer and focal retro-orbital uptake on scintigraphy (not shown here). Axial T2W (**a**), axial fluid attenuation inversion recovery (**b**), sagittal T1W (**c**), and axial T1W fat-saturated CE (**d**) MRI images show a hypervascular clival metastasis (white arrows)

Sphenoid bone metastases (Fig. 19) are very rare.﻿ Prostate adenocarcinoma, thyroid carcinoma, hepatocarcinoma, and breast cancer are the most common histological subtypes of tumour [101]. Sphenoid metastatic localization is almost often a late manifestation in multi-metastatic patients. Cancer cells reach the sphenoid and clivus mainly by venous route, reaching the basilar plexus in two different ways: through the Batson’s plexus, where the blood from the thoraco-abdominal district passes during the Valsalva manoeuvre, bypassing the caval venous system, or through the inferior petrosal sinus, which drains from the facial and ocular districts [102]. CT and MRI features are not specific and they may mimic a primary tumour.

*Differential diagnoses*: all the possible clival malignant neoplasms, especially chordoma, chondrosarcoma and plasmacytoma [102]. As already stated, CT/MRI features are not specific for metastasis; thus, a properly collected past history and the presence of multiple similar alterations in the cranio-facial area or elsewhere are necessary for the diagnosis [101].

## Conclusions

The sphenoid bone and the clivus can be primarily or secondarily affected by a large spectrum of pathologic conditions. CT and MRI are nowadays the reference imaging techniques to characterise sphenoid and clival abnormalities. Both techniques are almost always necessary to study these bones since they are scarcely susceptible to clinical assessment. Furthermore, any invasive diagnostic or therapeutic procedure has to be carefully evaluated because of its delicate anatomical relationships. For these reasons, radiologists have the crucial task to:

- 1) Identify the sphenoid/clival lesions that can be characterised with certainty by imaging techniques such as the “do-not-touch lesions”.
- 2) Provide a list of differential diagnosis between inflammatory and neoplastic lesions in order to identify or rule out malignancies.
- 3) Cooperate with skull base surgeons and pathologists to reach the correct diagnosis of those lesions that may be characterised by imaging techniques alone.

We are confident that, with a sound knowledge of imaging features, a correct non-invasive identification of lesions arising in this complex area will be possible in most cases.

## Data Availability

All data provide in the submitted work come from University Hospital of Careggi in Florence. All data are made available to the editors and reviewers.

## Abbreviations

**MRI**: Magnetic resonance imaging
**CT**: Computed tomography
**SI**: Signal intensity
**CE**: Contrast enhancement
**DWI**: Diffusion weighted imaging
**ADC**: Apparent diffusion coefficient

## Code availability

No specific software was used to carry out the submitted work.
